# The Effect of Substituent, Degree of Acetylation and Positioning of the Cationic Charge on the Antibacterial Activity of Quaternary Chitosan Derivatives

**DOI:** 10.3390/md12084635

**Published:** 2014-08-21

**Authors:** Priyanka Sahariah, Vivek S. Gaware, Ramona Lieder, Sigríður Jónsdóttir, Martha Á. Hjálmarsdóttir, Olafur E. Sigurjonsson, Már Másson

**Affiliations:** 1 Faculty of Pharmaceutical Sciences, School of Health Sciences, University of Iceland, Hofsvallagata 53, IS-107 Reykjavík, Iceland; E-Mails: prs1@hi.is (P.S.); vsg3@hi.is (V.S.G.); 2 PCI Biotech AS, Strandveien 55, N-1366 Lysaker, Norway; 3 The REModeL Lab, The Blood Bank, Landspitali University Hospital, Snorrabraut 60, 105 Reykjavik, Iceland; E-Mails: ramona@landspitali.is (R.L.); oes@landspitali.is (O.E.S.); 4 Department of Chemistry, Science Institute, University of Iceland, Dunhagi 3, IS-107 Reykjavik, Iceland; E-Mail: sigga@hi.is; 5 Department of _Biomedical Science, Faculty of_ Medicine, University of _Iceland, Stapi, Hringbraut 31, 101 Reykjavík, Iceland; E-Mail: hjalmars@hi.is; 6 Institute of Biomedical and Neural Engineering, Reykjavik University, Menntavegur 1, 101 Reykjavik, Iceland

**Keywords:** silyl chitosan, trimethyl chitosan (TMC), quaternary ammoniumyl, pyridiniumyl derivatives, antibacterial activity, structure-activity relationship (SAR)

## Abstract

A series of water-soluble cationic chitosan derivatives were prepared by chemoselective functionalization at the amino group of five different parent chitosans having varying degrees of acetylation and molecular weight. The quaternary moieties were introduced at different alkyl spacer lengths from the polymer backbone (C-0, C-2 and C-6) with the aid of 3,6-di-*O*-*tert*-butyldimethylsilyl protection of the chitosan backbone, thus allowing full (100%) substitution of the free amino groups. All of the derivatives were characterized using ^1^H-NMR, ^1^H-^1^H COSY and FT-IR spectroscopy, while molecular weight was determined by GPC. Antibacterial activity was investigated against Gram positive *S. aureus* and Gram negative *E. coli*. The relationship between structure and activity/toxicity was defined, considering the effect of the cationic group’s structure and its distance from the polymer backbone, as well as the degree of acetylation within a molecular weight range of 7–23 kDa for the final compounds. The *N*,*N*,*N*-trimethyl chitosan with 100% quaternization showed the highest antibacterial activity with moderate cytotoxicity, while increasing the spacer length reduced the activity. Trimethylammoniumyl quaternary ammonium moieties contributed more to activity than 1-pyridiniumyl moieties. In general, no trend in the antibacterial activity of the compounds with increasing molecular weight or degree of acetylation up to 34% was observed.

## 1. Introduction

Chitin is a structural polysaccharide that forms the basic constituent of the outer skeleton of insects and crustaceans, including shrimps and crabs. Chitin can be partially or fully deacetylated using strong alkali to give chitosan. Chitosan is therefore a heteropolysaccharide comprised of 2-amino-2-deoxy-d-glucopyranose (glucosamine) and *N*-acetyl glucosamine units linked through (1→4)-β-glycosidic bonds. A number of applications have been found for chitosan in the fields of pharmaceutics [1], biomedicine [2], cosmetics [3] and the food industry [4], due to its unique combination of various properties, like bioactivity, biocompatibility, biodegradability and lack of toxicity [5,6].

Amongst its various properties, the antimicrobial efficacy and applications of chitosan against bacteria have been the focus of many investigations. Chitosan has limited solubility in aqueous media above pH 6. It shows antibacterial properties only in acidic media. This activity is not observed at high pH, due to the absence of the positively charged amino groups and also due to low solubility in aqueous media [7,8,9]. Chitosan derivatives, in which permanent positive charges were introduced onto the polymer backbone, have been synthesized, which led, in general, to good aqueous solubility and also contributed to significant antibacterial activity at neutral pH [10]. Previously, such derivatives have been prepared by quaternizing the amino group of native chitosan [11,12] or by introducing the quaternized group in one step through an acylation or alkylation reaction [13,14]. This leads to products that are heterogeneous with respect to the degree of substitution (DS) on the amino group and often partially *O*-modified [15]. Regioselective triphenylmethyl (trityl) protection of the primary (C-6) hydroxyl group of chitosan to give 6-*O*-trityl chitosan has also been utilized to facilitate the synthesis of *N*-chloroacyl [16,17], *N*-betaine [18] and quaternary piperazine derivatives of chitosan [19,20]. Although the use of such selective protection resulted in higher DS, this led to an increase in the number of synthetic steps, and some modification at unprotected hydroxyl groups can also be observed [21]. Recently, we reported on silyl protected 3,6-di-*O*-*tert*-butyldimethylsilylchitosan (diTBDMS-CS) [22,23], which has been utilized in various chemoselective modifications to give products like *N*-(bromoacetyl)-diTBDMS-chitosan, *N*-(2-(*N*,*N*,*N*-trialkylammoniumyl)-chitosan, *N*,*N*,*N*-trimethyl chitosan and chitosan derivatives modified by covalent linking of the highly lipophilic photosensitizer, *meso*-tetraphenylporphyrin [24,25]. The TBDMS-protected precursor enabled the synthesis to be carried out in an organic medium, thereby allowing well controlled and regioselective modification, leading to homogenous products that can be fully characterized by spectroscopy with techniques, such as ^1^H-NMR, FT-IR, COSY and HSQC.

The role of the cationic charge in the antimicrobial effect is believed to be associated with the binding of the polymer to the bacterial cell wall. Several models have been proposed to explain the antimicrobial activity of chitosan, but the most accepted is electrostatic interaction between the positive charges on the polymer and the negatively charged anionic components of the bacterial surface, which weakens the cell wall and leads to cell lysis [26]. The polycationic structure of chitosan is a pre-requisite for antibacterial activity in spite of the structural differences in Gram positive and Gram negative bacteria [27]. Removal of the cell wall brings the polymer in contact with the cell membrane, thereby affecting membrane permeability and even reversing the surface charge of the bacteria [28]. These reactions finally lead to the leakage of the intracellular components, as evidenced by increased absorption at 260 nm [28], the increased electrical conductivity of the cell suspension [29] and cytoplasmic β-galactosidase release [30,31,32,33].

The structure-activity relationship (SAR) for chitosan and chitosan derivatives is not well understood. The relation between molecular weight (Mw) and degree of acetylation (DA) of chitosan to its antibacterial properties has also been explored. While high Mw and degree of quaternization (DQ) of *N*,*N*,*N*-trimethyl chitosan chloride (TMC) derivatives showed high bactericidal activity against both *S. aureus* and *E. coli* [34], in another study, it was reported that low Mw chitosan and its derivatives showed better activity [35,36]. A lower DA of acid-soluble chitosan was shown to lead to a greater inhibitory effect against *S. aureus* and *E. coli* [37,38,39], while some other studies have not shown a clear relationship between DA and the antimicrobial effect of unmodified chitosan [40,41].

In the current study, we used five different parent chitosan materials with variations in DA and Mw. These materials were used to synthesize different *N*-modified alkyl quaternary ammoniumyl and pyridiniumyl chitosan derivatives, such as (trimethylammoniumyl)acetyl, (trimethylammoniumyl)hexanoyl, (1-pyridiniumyl)acetyl, (1-pyridiniumyl)hexanoyl and *N*,*N*,*N*-trimethyl chitosan. These quaternary chitosan derivatives were then investigated for their antibacterial effects to allow systematic investigation of SAR under conditions where the effect of the functional group and the spacer length, as well as variations in the activity with the Mw and DA of the chitosan could be observed.

## 2. Results and Discussion

The quaternary ammoniumyl and 1-pyridiniumyl derivatives were synthesized from five different chitosan parent materials (denoted in superscript, e.g., **i**–**v**) (CS**^i^**^–**v**^) varying in their DA from 6% to 34% and from 180 to 308 kDa in their Mw.

The quaternary groups were distanced from the polymer backbone with alkyl chain spacers. Each spacer length required a different approach to the synthesis; the discussion on the synthesized derivatives is therefore divided into four sections in accordance with the length of the alkyl chain (C-spacer) or its absence.

### 2.1. Synthesis of N-(2-(N,N,N-Trimethylammoniumyl)acetyl)-chitosan Chloride (TMA-CS) and N-(2-(1-Pyridiniumyl)acetyl)-chitosan Chloride (PyA-CS), the C-2 Spacer Chitosan Derivatives

**Scheme 1:**
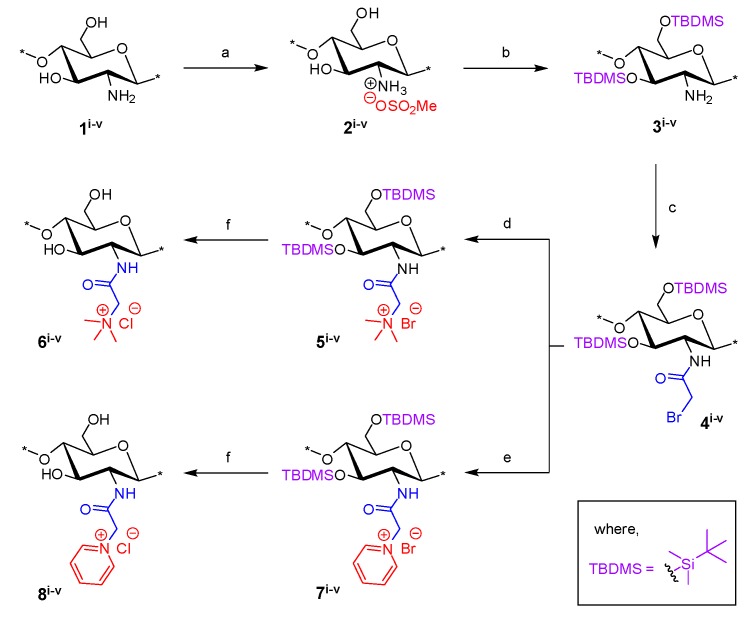
Synthesis of final *N*-(2-(*N*,*N*,*N*-trimethylammoniumyl)acetyl)-chitosan chloride (TMA-CS) (**6^i^**^–**v**^) and *N*-(2-(1-pyridiniumyl)acetyl)-chitosan chloride (PyA-CS)(**8^i^**^–**v**^) derivatives. Reactions and conditions: (**a**) MeSO_3_H/H_2_O (1:1), 10 °C, 1 h (90%); (**b**) tert-butyl-dimethylsilyl chloride (TBDMSCl), imidazole, DMSO, 25 °C, 24 h (96%); (**c**) bromoacetyl bromide, Et_3_N, CH_2_Cl_2_, −20 °C, 1 h (92%); (**d**) Me_3_N (31%–35% wt in EtOH, 4.2 M),_CH_2_Cl_2_,_25 °C, 12 h; (**e**) pyridine, 25 °C, 24 h; (**f**) conc HCl/MeOH, 25 °C, 24 h, ion exchanged by (8%) acqueos NaCl (w/v), 1 h, dialysed against de-ionised water, 48h.

The synthetic route to prepare the final TMA-CS (**6^i^**^–**v**^) and PyA-CS (**8^i^**^–**v**^) is shown in Scheme 1. Initially, all five different chitosan materials (**1^i^**^–**v**^) were converted to their corresponding chitosan mesylate salts (Mes-CS) (**2^i^**^–**v**^) by careful dropwise addition of methanesulfonic acid to the chitosan suspended in water at 10 °C. The finely powdered materials (**2^i^**^–**v**^**)** were obtained by following our earlier reported protocol. Unlike chitosan starting materials (**1^i^**^–**v**^), these mesylates, **2^i^**^–**v**^, were completely soluble in H_2_O, as well as in organic solvents, such as DMSO. The solubility of Mes-CS in DMSO was important, as it facilitated quantitative silyl protection of both hydroxyl groups on the CS under homogeneous conditions. Fully silyl-protected diTBDMS-CS (**3^i^**^–**v**^) materials were then obtained by using tert-butyl-dimethylsilyl chloride (TBDMSCl) and imidazole in DMSO at 25 °C. The intermediate *N*-(bromoacetyl)-3,6-di-*O*-TBDMS-chitosan (BrA-diTBDMS-CS) (**4^i^**^–**v**^) was prepared by reacting silyl chitosan **3^i^**^–**v**^ with four equivalents of bromoacetyl bromide in the presence of five equivalents of triethylamine (Et_3_N). The reaction temperature was carefully maintained at −20 °C throughout the reaction, and the reaction was quenched after 1 h to avoid any side reactions. The crude material was washed with acetonitrile (CH_3_CN) to afford the fine powdered material, which was completely soluble in dichloromethane (CH_2_Cl_2_). Freshly prepared reactive intermediate **4^i^**^–**v**^ was then reacted at 25 °C in CH_2_Cl_2_, with an excess of NMe_3_ or pyridine to afford compounds **5^i^**^–**v**^ and **7^i^**^–**v**^, respectively. Compounds **5^i^**^–**v**^ and **7^i^**^–**v**^ were finally deprotected using concentrated (conc) HCl/MeOH to afford the corresponding final quaternized chitosan derivatives, **6^i^**^–**v**^ and **8^i^**^–**v**^, respectively (Scheme 1).

**Figure 1:**
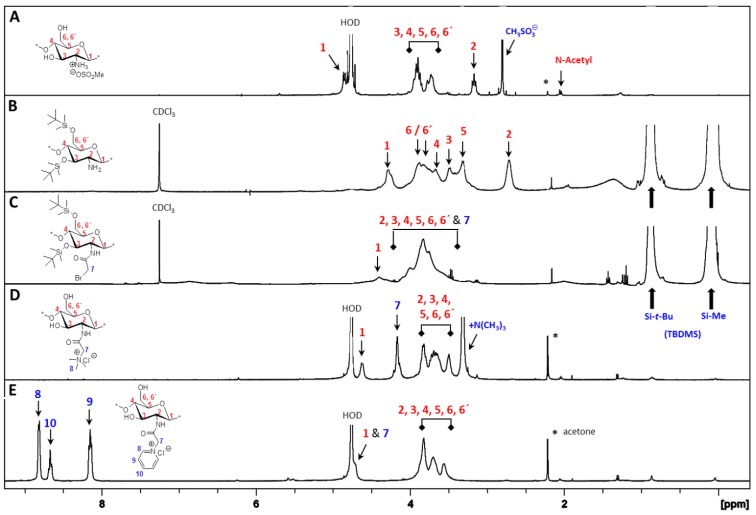
^1^H NMR spectra overlay of the main compounds and final C-2 spacer quaternary derivatives of the representative chitosan material (CS-i, 7%_DA): (**A**) chitosan mesylate salts (Mes-CS), **2^i^**; (**B**) diTBDMS-CS, **3^i^**; (**C**) *N*-(bromoacetyl)-3,6-di-*O*-TBDMS-chitosan (BrA-diTBDMS-CS), **4^i^**; (**D**) TMA-CS, **6^i^**; (**E**) PyA-CS, **8^i^**.

**Figure 2:**
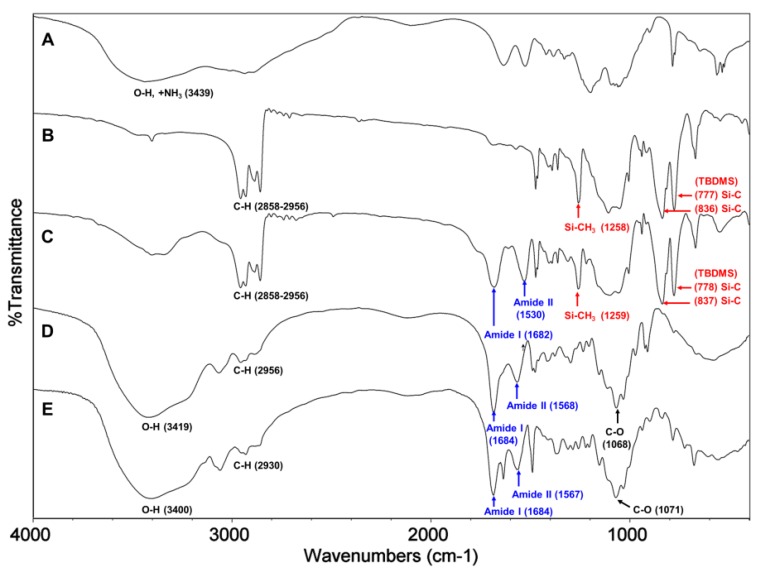
FT-IR spectra overlay of the main compounds and final C-2 spacer quaternary derivatives of the representative chitosan material (CS-i, 7%_DA): (**A**) Mes-CS, **2^i^**; (**B**) diTBDMS-CS, **3^i^**; (**C**) BrA-diTBDMS-CS, **4^i^**; (**D**) TMA-CS, **6^i^**; (**E**) PyA-CS, **8^i^**.

^1^H NMR and FT-IR analysis. All of the key intermediates and final TMA-CS and PyA-CS derivatives were thoroughly characterized. The ^1^H NMR (Figure 1) and FT-IR (Figure 2) overlay comparison of chitosan derivatives at different stages of synthesis confirmed the *N*-selective covalent modification with 100% substitution at the free amino groups. ^1^H NMR and IR spectra of silyl chitosan (**3^i^**) and the bromoacyl intermediate (**4^i^**) showed complete silyl protection of both hydroxyls (C-3 and C-6). ^1^H NMR (Figure 1B,C) and FT-IR of **4^i^** (Figure 2C) showed characteristic amide bond peaks (1676, 1527 cm^−1^) with no sign of ester functionality, as expected for *N*-selective modification. Characteristic TBDMS peaks indicated by red arrows at 1259, 778 and 837 cm^−1^ and C-H peaks at 2858–2956 cm^−1^ remained intact. The final derivatives, TMA-CS (**6^i^**) and PyA-CS (**8^i^**), were completely soluble in D_2_O (Figure 1D,E) after the removal of the TBDMS peaks, as seen by their absence in the NMR and FT-IR spectra (Figure 2D,E).

**Figure 3:**
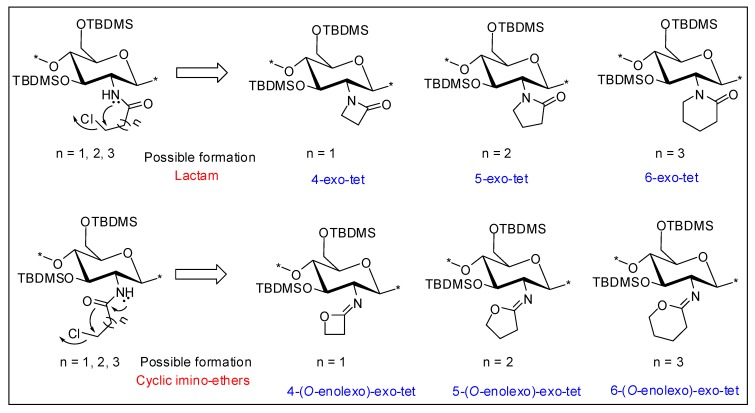
Possible intramolecular cyclization of C-3, C-4 and C-5 spacer compounds based on the findings of Stirling *et al.* [43].

Synthesis of C-3 and C-5 spacer chitosan derivatives: An attempt to synthesize C-3 and C-5 spacer derivatives using 3-chloropropionyl chloride and 5-chlorovaleroyl chloride, respectively, under similar conditions did not succeed. This may be explained by the formation of stable four- or six-membered ring compounds by the intermediate (Figure 3), which will be favored according to Baldwin’s rules for favorable ring closure of four-, five- or six-membered rings [42]. Stirling *et al.* (1960) have reported the mechanism of similar intramolecular cyclization of bromo-amides under basic or neutral conditions [43]. The cyclization was indicated in the ^1^H NMR spectra of the chloroacyl-intermediates, where the final deprotected material having the C-3 and C-5 spacer was not soluble in water, indicating that the desired quaternized product was not obtained. The FT-IR analysis (data not shown) of these materials also indicated ring fusion (Figure 3), similar to what was described by Stirling *et al.* [43].

### 2.2. Synthesis of N-(6-(N,N,N-Trimethylammoniumyl)hexanoyl)-chitosan Chloride (TMHA-CS) and N-(6-(1-Pyridiniumyl)hexanoyl)-chitosan Chloride (PyHA-CS), C-6 Spacer Derivatives

**Scheme 2:**
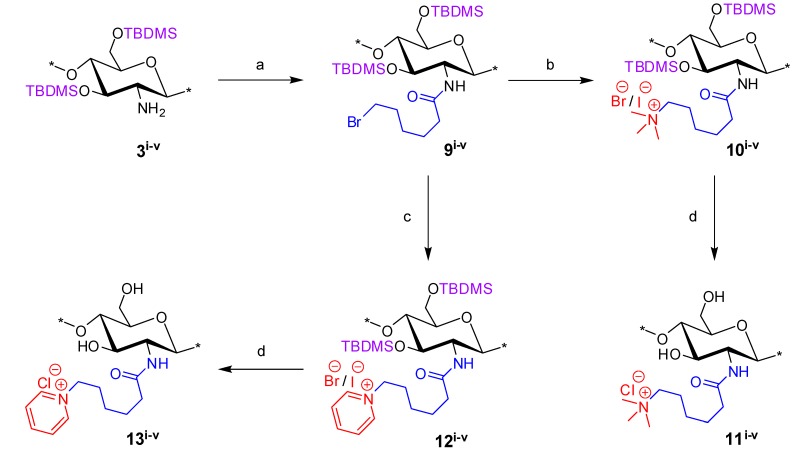
Synthesis of final *N*-(6-(*N*,*N*,*N*-trimethylammoniumyl)hexanoyl)-chitosan chloride (TMHA-CS) (**11^i^**^–**v**^) and PyHA-CS (**13^i^**^–**v**^) derivatives. Reactions and conditions: (**a**) 6-bromohexanoyl chloride, Et_3_N, CH_2_Cl_2_, −20 °C, 1 h (69%); (**b**) Me_3_N (31%–35% wt in EtOH, 4.2 M), KI, CH_2_Cl_2_, 25 °C, 48 h; (**c**) pyridine, KI, 25 °C, 48 h; (**d**) conc HCl/MeOH, 25 °C, 24 h, ion exchange by (5%–8%) NaCl (aqueous) (w/v), 1 h, dialysed against de-ionised water, 48 h.

The derivatives, *N*-(6-(*N*,*N*,*N*-trimethylammoniumyl)hexanoyl)-chitosan chloride (TMHA-CS) (**11^i^**^–**v**^) and PyHA-CS (**13^i^**^–**v**^), were synthesized (Scheme 2) under conditions similar to those used for the C-3 spacer compounds, with slight modifications. The key electrophilic intermediate, **9^i^**^–**v**^, was prepared by reacting silyl chitosan **3^i^**^–**v**^ with four equivalents of 6-bromohexanoyl_chloride in the presence of five equivalents of Et_3_N at −20 °C for 1 h, under almost identical conditions as those described for similar key intermediates with the C-2 spacer, *i.e.*, **4^i^**^–**v**^. Intermediate **9^i^**^–**v**^ was also completely soluble in CH_2_Cl_2_. However, unlike intermediate **4^i^**^–**v**^, **9^i^**^–**v**^ was found to be more stable and, hence, need not be used immediately after its preparation. The intermediate **9^i^**^–**v**^ was confirmed by ^1^H NMR and FT-IR analysis. Intermediate **9^i^**^–**v**^ when reacted with an excess of NMe_3_ or pyridine, afforded the corresponding compounds **10^i^**^–**v**^ and **12^i^**^–**v**^, respectively. However, the reaction with NMe_3_ and/or pyridine appeared to be slower in case of the C-6 spacer as compared to the shorter spacer (C-2), and thus, potassium iodide (KI) was used as a catalyst to assist the reaction along with prolonged reaction time. Crude compounds **10^i^**^–**v**^ and/or **12^i^**^–**v**^ were then subjected to final deprotection with conc HCl/MeOH at 25 °C, and the materials were ion-exchanged using aqueous NaCl (5%–8%) (w/v), dialyzed and freeze dried to afford the corresponding final TMHA-CS (**11^i^**^–**v**^) and PyHA-CS (**13^i^**^–**v**^) derivatives, respectively. The trimethylammoniumyl derivatives solubilized faster in water compared to the pyridinium derivatives.

**Figure 4:**
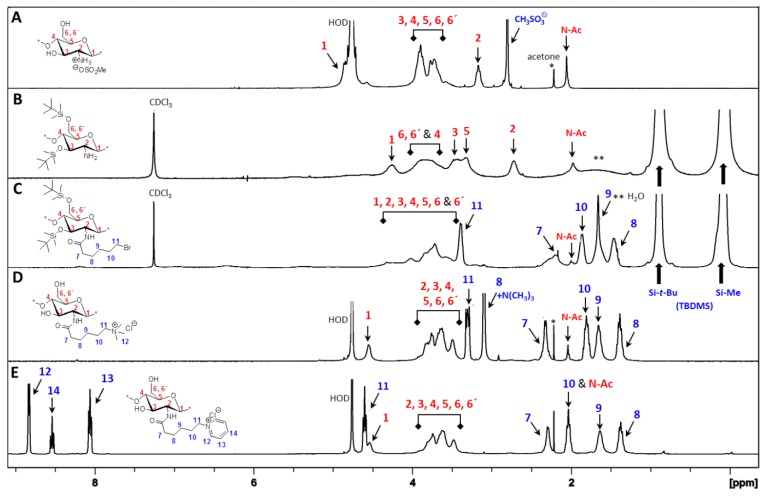
^1^H NMR spectra overlay of the main compounds and final C-6 spacer quaternary derivatives of the representative chitosan material (CS-iv, 19%DA): (**A**) Mes-CS (**2^iv^**); (**B**) diTBDMS-CS (**3^iv^**); (**C**) BrHA-diTBDMS-CS (**9^iv^**); (**D**) TMHA-CS (**11^iv^**); (**E**) PyHA-CS (**13^iv^**).

**Figure 5:**
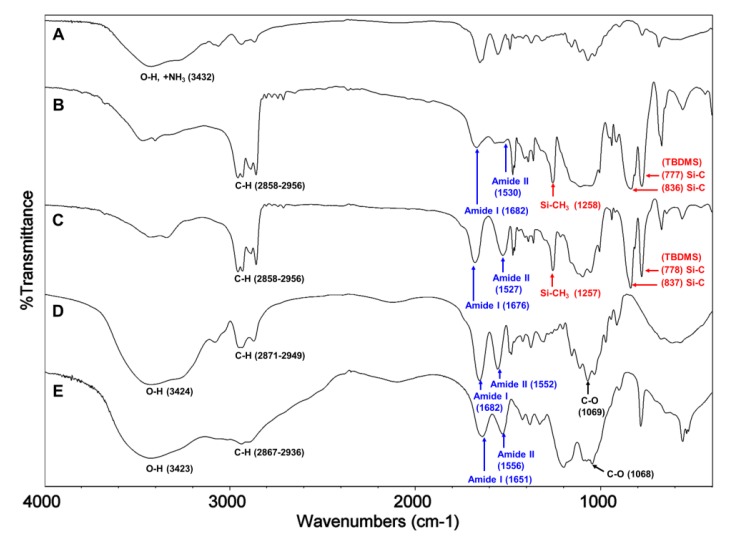
FT-IR overlay of the main compounds and final quaternary derivatives of the representative chitosan material (CS-iv, 19%_DA): (**A**) Mes-CS (**2^iv^**); (**B**) diTBDMS-CS (**3^iv^**); (**C**) BrHA-diTBDMS-CS (**9^iv^**); (**D**) TMHA-CS (**11^iv^**); (**E**) PyHA-CS (**13^iv^**).

^1^H NMR and FT-IR analysis: Synthesis of the C-6 spacer compounds was followed by ^1^H NMR (Figure 4) and FT-IR (Figure 5) analysis. In the ^1^H NMR of diTBDMS-CS (**3^iv^**) in CDCl_3_ (Figure 4B), the broadening of individual backbone peaks could be seen. This can be attributed to the increased viscosity of the material. The ^1^H NMR spectra of BrHA-diTBDMS-CS (**9^iv^**) (Figure 4C) showed that the backbone peaks (H-1 to H-6′) appeared together while the CH_2_ peaks of the alkyl chain, *N*-acetyl peak and TBDMS peaks could be assigned individually. Furthermore, FT-IR spectra of **3^iv^** (Figure 5B) and **9^iv^** (Figure 5C) confirmed the characteristic TBDMS peaks (shown in red arrows) and amide peaks (in blue arrows). The final C-6 spacer derivatives, TMHA-CS (**11^iv^**) (Figure 4D) and PyHA-CS (**13^iv^**) (Figure 4E) could also be confirmed by their individual distinct peaks. The spectra confirmed 100% substitution of the amino groups by either trimethylammoniumyl or 1-pyridiniumyl moieties. The IR spectra (Figure 5D,E) also confirmed the deprotection.

### 2.3. Synthesis of C-0 Spacer TMC Derivatives (**15^i–v^**)

**Scheme 3:**
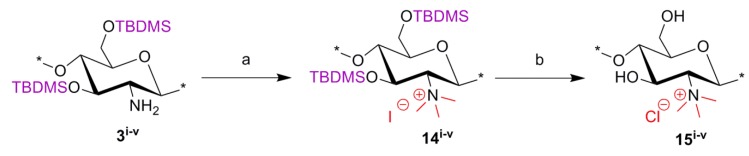
Synthetic route for *N*,*N*,*N*-trimethyl chitosan chloride (TMC) derivatives (**15^i^**^–**v**^). Reactions and conditions: (**a**) CH_3_I, Cs_2_CO_3_, NMP, 45–50 °C; (**b**) TBAF (1 M), NMP, 50 °C.

*N*,*N*,*N*-trimethyl-chitosan (TMC) was synthesized according to the procedure developed by Benediktsdottir *et al.* [25] as shown in Scheme 3. Briefly, diTBDMS-CS (**3^i^**^–**v**^) was dispersed in *N*-methyl-2-pyrrolidone (NMP), and methylation was carried out using methyl iodide (CH_3_I) in the presence of cesium carbonate (Cs_2_CO_3_) as a base. This method resulted in 100% trimethyl substitution at the amino group without any *O*-methylation. The excess CH_3_I and NMP used in the reaction were removed by dialyzing the material against deionized water followed by freeze-drying. The *N*,*N*,*N*-trimethyl-diTBDMS-CS iodide (**14^i–iv^**) was then subjected to deprotection of the silyl groups under 1M tetrabutyl ammonium fluoride (TBAF)/NMP solution. In cases where traces of the silyl groups still remained in the polymer, the deprotection was repeated under the same conditions. The products were then characterized using ^1^H-NMR, ^1^H-^1^H COSY and IR spectra. The appearance of a new peak at 3.64 ppm in *N*,*N*,*N*-trimethyl-diTBDMS-CS indicated the trimethyl substitution in the polymer. However, due to the presence of the diTBDMS groups, the peaks were broadened and the exact DS was difficult to determine. Only after removal of the silyl groups did the polymer peaks became sharper, so the DS could be calculated from the integrals of H-1 (5.49 ppm) and the trimethyl (3.35 ppm) peak.

### 2.4. Physicochemical Properties of Chitosan-Derivatives

The DA was calculated by ^1^H NMR analysis of the parent chitosan materials. The weight average molecular weight (Mw) of parent chitosan materials, Mes-CS and the final quaternized derivatives of the chitosan were determined by gel permeation chromatography (GPC) (Table 1).

**Table 1:** Physical properties of chitosan derivatives.

Parent Chitosan Material	DA (%)	Chitosan (1^i–v^)		Mes-CS (2^i–v^)		TMA-CS (6^i–v^)		PyA-CS (8^i–v^)		TMHA-CS (11^i–v^)		PyHA-CS (13^i–v^)		TMC (15^i–v^)	
		Mw	(PDI)	Mw	(PDI)	Mw	(PDI)	Mw	(PDI)	Mw	(PDI)	Mw	(PDI)	Mw	(PDI)
CS-i	7	235	(2.8)	24.6	(1.6)	23.8	(2.1)	18.8	(1.8)	17.3	(1.6)	12.9	(1.5)	18.7	(1.5)
CS-ii	6	294	(2.3)	20.8	(1.9)	17.1	(1.8)	17.3	(2.0)	15.1	(1.5)	9.8	(1.3)	15.3	(1.4)
CS-iii	17.3	225	(2.6)	19.1	(1.6)	16.4	(1.6)	12.2	(1.9)	13.1	(1.4)	-	-	13.2	(1.4)
CS-iv	19	308	(2.6)	21.4	(2.4)	16.7	(1.6)	14.6	(1.8)	14.2	(1.8)	15.8	(1.1)	19.8	(1.6)
CS-v	34.2	180	(2.9)	19.5	(1.5)	18.9	(1.7)	10.8	(1.4)	7.4	(1.4)	-	-	16.5	(1.5)

The average molecular weight (Mw) is in kDa, and the polydispersity index is abbreviated as (PDI). DA = degree of acetylation.

Though the parent chitosan (CS-i to CS-v, **1^i^**^–**v**^) has a higher range of Mw (180–308 kDa), significant degradation of the polymer chain was observed after their conversion to the corresponding mesylate salts (**2^i^**^–**v**^) in all five materials, as this step requires highly acidic conditions, which are known to cause hydrolysis of glycosidic bonds in the polymer backbone [44]. Any further degradation after the first step was limited; so, although the Mw values were reduced, the relative range of Mw (7–23 kDa) for the derivatives was comparable to that of the starting chitosan materials.

### 2.5. Antibacterial Properties

The antibacterial activities of the quaternary chitosan derivatives were determined by measuring the minimum inhibitory concentration (MIC) and minimum lethal concentration (MLC) values against *S. aureus* and *E. coli*. The derivatives varied in their quaternary group, in the length of the spacer bearing the quaternary group, Mw and also in the DA of the chitosan. These parameters were used to observe the influence of the chitosan polymer on the antibacterial properties in order to develop an overall structure-activity relationship. The quaternary derivatives carrying positive charges were highly soluble in water, and hence, their activity could be tested at neutral pH. Table 2 shows that the chitosan derivatives exerted an antibacterial effect against both bacterial strains, with *S. aureus* yielding lower MIC and MLC values compared to *E. coli*. With respect to the variation in the functional group and the length of the spacer carrying the functional group, the following trend in activity was observed: TMC derivatives (**15^i^**–**15^v^**) exhibited the highest antibacterial effect against both strains, with MIC values ranging from 4 to 32 μg/mL against *S. aureus* and 64 to 256 μg/mL against *E. coli*, respectively. These results were consistent with earlier studies where TMC (DS = 0.86) was found to have a MIC of 8 μg/mL and 128 μg/mL against *S. aureus* and *E. coli*, respectively [45]. Thus, the degree of trimethylation does not seem to have a pronounced effect on antibacterial activity, as the DS increased from 0.86 to 0.95. The activity of the series of TMA-CS derivatives (**6^i^**–**6^v^**) was comparable to that of the TMC derivatives against *S. aureus*, but was much lower against *E. coli*. These derivatives showed similar activity as the previously synthesized TMA-CS derivatives (DS = 0.8; MIC = 128 and ≥8192 μg/mL against *S. aureus* and *E. coli*, respectively) [45]. The TMHA-CS derivatives (**11^i^**–**11^v^**) with a C-6 spacer had still lower activity against *S. aureus* (MIC = 1024–2048 μg/mL) compared to the other two series carrying the same quaternary group. However, their activity was found to be higher than that of the TMAC-CS derivatives against *E. coli* (MIC = 128–1024 μg/mL). Thus, in most cases, a decreasing effect on activity could be observed as the spacer between the trimethylammonium group and the polymer increased. The second functional group, *i.e.*, a pyridinium moiety attached to polymers having an acetyl (PyA-CS) or a C-6 spacer (PyHA-CS), also showed bactericidal activity against *S. aureus*; however, they were found to be less effective than their corresponding trimethylammonium derivatives. The acetyl pyridinium moiety exhibited MIC values of 512–1024 μg/mL against *S. aureus*, while the activity against *E. coli* was lower, with the MIC ranging from 128 to 16,384 μg/mL. The C-6 spacer derivative, as expected, showed still less activity than the acetyl pyridinium derivatives against both strains. They were active only against *S. aureus* (MIC = 2048–8192 μg/mL) while remaining almost inactive against the Gram negative *E. coli* within the range of concentrations measured. These results are in agreement with our previous investigations, where our results indicated that antimicrobial action was more efficient when the cationic charge is located at the amino group of the chitosan and not on the quaternary substituent [46].

**Table 2:** Antibacterial activity, hemolytic activity and cytotoxicity of the quaternary chitosan derivatives.

Compounds	Structure	*S. aureus* (ATCC 29213)		*E. coli* (ATCC 25922)		HC_50_ (μg/mL)	Selectivity (HC_50_/MIC)		EC_50_ (μg/mL)
		MIC (μg/mL)	MLC (μg/mL)	MIC (μg/mL)	MLC (μg/mL)		*S. aureus*	*E. coli*	
TMC (**15^i^**)	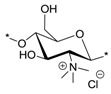	8	64	256	256	6114	764	47.7	40
TMC (**15^ii^**)		32	32	64	64	6114	191	95.5	-
TMC (**15^iii^**)		4	4	64	64	6114	1528	95.5	-
TMC (**15^iv^**)		8	8	256	256	3072	764	47.7	10
TMC (**15^v^**)		32	32	256	1024	640	191	-	-
TMA-CS (**6^i^**)	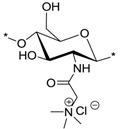	8	8	16,384	≥32,768	≥32,768	≥4096	≥2	26
TMA-CS (**6^ii^**)		8	8	16,384	16,384	≥32,768	≥4096	≥2	-
TMA-CS (**6^iii^**)		32	32	16,384	16,384	≥32,768	≥1024	≥2	-
TMA-CS (**6^iv^**)		32	32	≥32,768	≥32,768	≥32,768	≥1024	-	66
TMA-CS (**6^v^**)		128	128	≥32,768	≥32,768	≥32,768	≥256	-	-
PyA-CS (**8^i^**)	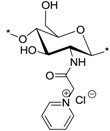	8	1024	16,384	16,384	≥32,768	≥8192	≥2	38
PyA-CS (**8^ii^**)		8	512	8192	8192	≥32,768	≥8192	≥4	-
PyA-CS (**8^iii^**)		1024	1024	16,384	16,384	≥32,768	≥8	≥2	-
PyA-CS (**8^iv^**)		512	1024	16,384	16,384	≥32,768	≥16	≥2	12
PyA-CS (**8^v^**)		512	512	128	8192	≥32,768	≥64	≥256	-
TMHA-CS (**11^i^**)	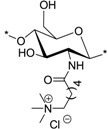	1024	2048	256	≥32,768	≥32,768	≥32	≥128	644
TMHA-CS (**11^ii^**)		2048	2048	512	16,384	≥32,768	≥16	≥64	-
TMHA-CS (**11^iii^**)		1024	2048	128	≥32,768	≥32,768	≥4	≥256	-
TMHA-CS (**11^iv^**)		2048	4096	512	≥32,768	≥32,768	≥8	≥64	108
TMHA-CS (**11^v^**)		1024	4096	1024	≥32,768	≥32,768	≥32	≥32	-
PyHA-CS (**13^i^**)	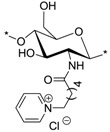	4096	4096	≥32,768	≥32,768	≥32,768	≥8	-	4
PyHA-CS (**13^ii^**)		2048	2048	≥32,768	≥32,768	≥32,768	≥32	-	-
PyHA-CS (**13^iii^**)		8192	8192	≥32,768	≥32,768	≥32,768	≥4	-	-
PyHA-CS (**13^iv^**)		2048	2048	16,384	16,384	≥32,768	≥16	≥2	18
PyHA-CS (**13^v^**)		2048	2048	≥32,768	≥32,768	≥32,768	≥16	-	-

The antibacterial tests was done according the Clinical and Laboratory Standards Institute (CLSI) protocol (see Section 3.4.1). According to this procedure, a single dilution series was done for each compound, and gentamycin was used as a positive control. A difference of 1–2 dilutions is therefore not considered significant. The hemolysis measurements were also done in singlets for each concentration. The cytotoxicity measurements were carried out in triplicate, and the standard deviation varied from 10% to 22%.

**Figure 6:**
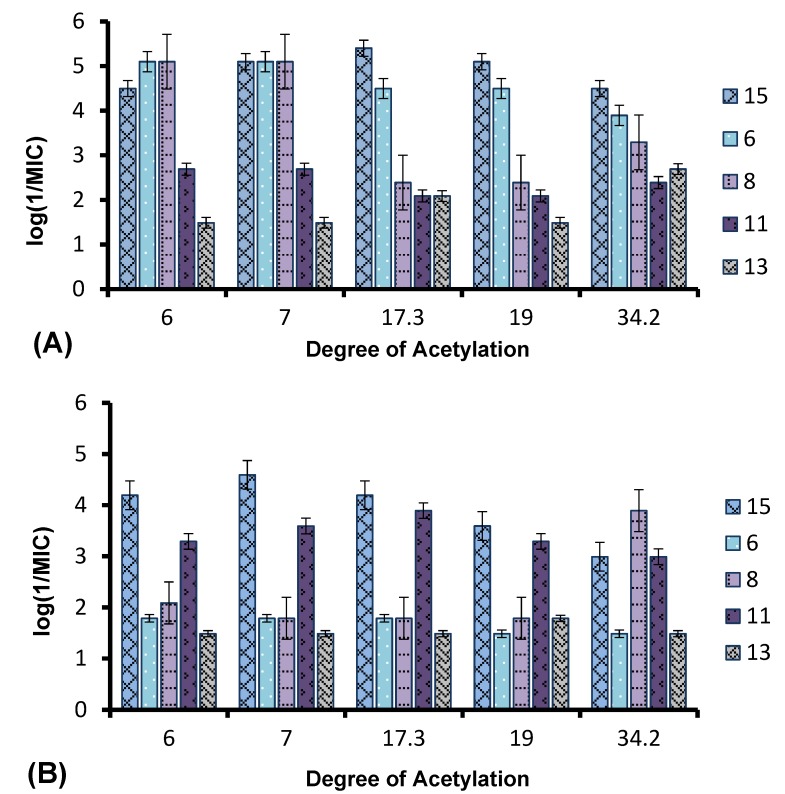
Variation in the antibacterial activity of chitosan with different DA against (**A**) *S. aureus* and (**B**) *E. coli*.

The effect of the DA and Mw of the chitosan on its antibacterial properties was also investigated. Chitosan derivatives carrying a particular functional group varied in their molecular weight and degree of acetylation. Although the staring chitosan samples had Mw variations from 180 to 308 kDa, due to degradations during the synthesis process, all of the derivatives showed considerably less Mw (7–23 kDa), but the relative range in Mw was not reduced. However, no trend in activity of the derivatives with variation in Mw could be observed. Hence, the difference was mainly based on the DA variation. Earlier studies reported different conclusions regarding the dependency of the antimicrobial activity of chitosan on the DA and Mw. One study of the antibacterial activity of chitosan indicated that low molecular weight chitosan (4.1–5.6 kDa) showed a greater inhibitory effect when the DA, ranging from 0.45–0.52, was reduced to 0.17–0.19 [47]. Again, in another study, the antibacterial activity was found to be independent of variation in the Mw (2–224 kDa) of chitosan, but to decline with increasing DA from 0.16 to 0.48 [48]. Figure 6A,B shows the variation in antibacterial activity (log 1/MIC) of the quaternary derivatives with DA against *S. aureus* and *E. coli*. In contrast, as seen in Figure 6B, only Series **8** showed an apparent increase in antibacterial activity (7–10-fold) against *E. coli* as the DA values increased from 19% to 34.2%. However when the MLC values are considered (Table 2), there is only one dilution difference in this series. For the Series **15** and **6**, the activity seemed to decrease gradually, as the DA changed from 6%–34% with only a 2–3-fold decrease in activity. In contrast, the activity of Series **11** and **13** remained independent of variations in DA against *S. aureus*. In Figure 6B, it can be observed that the activities of the compounds differed by only 1–2 dilutions, and no clear variation in activity with increasing or decreasing DA was observed. Thus, the activity of the complete series against *E. coli* remained independent of variations in DA.

The error bars indicate a deviation equivalent to one dilution.

The screening of the quaternary compounds against the two strains of bacteria and their results led us to the conclusion that the antibacterial activity of the chitosan derivatives decreased as the distance of the positive charge from the polymer backbone increased. This was observed for the trimethylammonium, as well as the pyridinium functional group. Thus, the antibacterial activity of the chitosan polymer not only depended on the number of positive charges present in the polymer, but also on the positioning of the positive charge on the polymer backbone. It has been earlier observed that the activities of other antimicrobial polymers with a synthetic backbone were dependent on the spacer length due to changes in the charge density and conformation of the polymer, which, in turn, affects its interaction with the bacterial membrane [49]. A study of the interaction of chitosan with model membranes has also shown that the mode of action of chitosan is not only related to electrostatic interactions, but also to its specific conformation in solution [50]. This may explain why the TMC derivatives showed the highest activity. In this case the positive charge was located on the polymer backbone, which may be favorable for the conformation required for binding to the bacterial membrane. However, as the spacer length increases, the polymer probably will tend to adopt a conformation that is less favorable for efficient binding with the anionic components of the bacterial membrane, and hence, the activity reduces. All of the quaternary chitosan derivatives showed greater activity, in general, against Gram positive *S. aureus* than Gram negative *E. coli*. This is in agreement with a previous observation where unmodified chitosan was shown to exhibit greater inhibitory effects against Gram positive bacteria as compared to Gram negative bacteria [51] due to differences in the cell wall structure [35]. In the case of Gram positive bacteria, the positive charges on chitosan were thought to bind to the thick peptidoglycan cell wall of the bacteria, resulting in cell wall disruption and leakage of the cellular contents. On the other hand, Gram negative bacteria possess an additional outer membrane composed of lipopolysaccharides, which provide the bacterium with a hydrophilic surface. The anionic units of the lipopolysaccharide form ionic type bonds with the cationic groups of the chitosan, which prevents nutrient flow and ultimately leads to the death of the cell due to depletion of nutrients [31,52]. Since the outer membrane serves as a penetration barrier against macromolecules and hydrophobic substances, overcoming the outer membrane barrier is a pre-requisite to displaying activity against Gram negative bacteria [29].

### 2.6. Hemolytic Activity and Cytotoxicity

**Figure 7:**
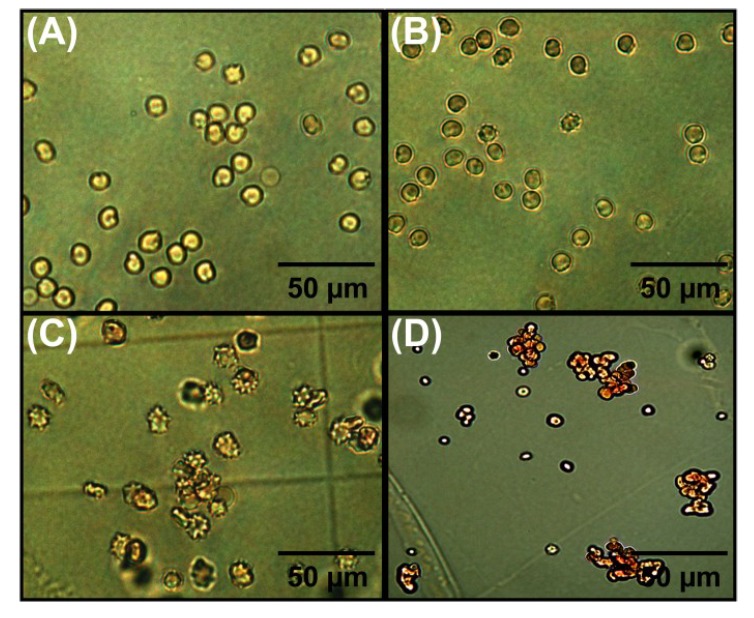
Light microscopic images of RBC. (**A**) RBC suspended in TBS; (**B**) RBC treated with Compound **15^iii^** (512 μg/mL); (**C**) RBC treated with Compound **15^iii^** (8192 μg/mL); and (**D**) RBC treated with 1% (v/v) Triton-X100.

To determine their toxicity, the compounds were tested for hemolytic activity against human red blood cells, and cytotoxicity was measured against the Caco-2 cell line. The antibacterial chitosan derivatives were not targeted for a specific organ, and hence, the compounds were evaluated only to get a general overview of potential toxicity. These compounds could be considered for surface treatments or as disinfectants and, therefore, were tested for hemolysis and the effect on a commonly used cell line (Caco-2) derived from mucosal epithelium. As seen in Table 2, the TMC derivatives (**15^i^**–**15^v^**), which showed the highest antibacterial activity, displayed low hemolytic activity ranging from 640 to 6114 μg/mL. The toxicity of the compounds against RBC decreased with decreasing DA of the derivatives within the series. However, no particular trend in HC_50_ values with Mw variations could be observed for the TMC series. Light microscopic images of red blood cells (RBC) treated with different concentrations of one polymer (**15^iii^**) are shown in Figure 7. Figure 7A shows a microscopic image of normal RBC suspended in TBS. When the RBCs were treated with different concentrations (512 μg/mL and 8,192 μg/mL) of the TMC derivative **15^iii^**, no hemolytic effect was observed with the lower concentration (512 μg/mL), as seen in Figure 7B, while the higher concentration (8192 μg/mL) produced the deformation of cell shape, as seen in Figure 7C. In Figure 7D, 100% hemolysis was observed with the release of hemoglobin when the cells were treated with the positive control Triton-X100. TMA-CS and some of the PyA-CS derivatives that showed comparable antibacterial activity to that of TMC did not show any hemolytic activity. The compounds in the other two series with the C-6 spacer also remained non-hemolytic within the measured concentration range. Thus, the ability of the polymers to lyse RBCs diminished as the quaternary group moved away from the polymer backbone.

The cell line cytotoxicity results, on the other hand, differed from those for hemolysis. TMA-CS showed cell toxicity at lower concentrations (10–40 μg/mL) compared to hemolysis. Compounds containing the trimethylammonium group with a spacer, *i.e.*, the TMA-CS (**6^i^**–**6^v^**) and TMHA-CS derivatives (**11^i^**–**11^v^**), were less cytotoxic compared to the TMC derivatives, while compounds containing the pyridinium group, *i.e.*, PyA-CS (**8^i^**–**8^v^**) and PyHA-CS (**13^i^**–**13^v^**), displayed cytotoxicity values comparable to those of TMC derivatives. No trend in the cytotoxicity of the compounds with changes in the length of the spacer could be observed. However, for most of the compounds, within a series, the toxicity was found to be lower in cases having a lower DA. The cytotoxicity was also found to be related to the Mw of the derivatives. Compounds having a lower Mw value within a series were found to exhibit lower cytotoxicity values. In many studies, antibacterial potency and an agent’s selectivity for bacteria over mammalian cells are quantified by determining the MIC and hemolytic activity (HC_50_ values) [53]. In our study, we saw that, although the compounds displayed low hemolytic activity, their cellular toxicity was found to be significant. Thus, in order to get a full picture of their potential toxicity, only testing compounds against erythrocytes is not sufficient, and more in-depth studies of potentially toxic effects against various cell types *in vitro* or *in vivo* should be a requirement.

## 3. Experimental Section

### 3.1. Materials

Five different parent chitosan materials provided by Genis ehf (Reykjavik, Iceland): (i) (CS-i (G030626-2) (Mw = 235 kDa, PDI = 2.8, DA = 0.07)); (ii) (CS-ii (S030626-2) (Mw = 294 kDa, PDI = 2.3, DA = 0.06)); (iii) (CS-iii (TM1238) (Mw = 225 kDa, PDI = 2.6, DA = 0.17)); (iv) (CS-iv (TM1534) (Mw = 308 kDa, PDI = 2.6, DA = 0.19)); and (v) (CS-v (S040108-1) (Mw = 180 kDa, PDI = 2.9, DA = 0.34)) were used for synthesis. All chemicals (procured from Sigma-Aldrich^®^) were used as received, except solvents, like DMSO, CH_2_Cl_2_ and NMP, which were stored over molecular sieves before use. Dialysis membranes obtained from Spectrum^®^ Laboratories Inc. (Breda, The Netherlands ,) (RC, Spectra/Por, Mw cutoff 3500 Da) and Float-A-Lyzers (Spectra/Por, Mw cutoff 3.5–5 kDa, 5-mL sample volume) were used for the dialysis of the final quaternary chitosan derivatives.

### 3.2. Characterization and Calculations

^1^H NMR, ^1^H-^1^H COSY samples were recorded with a Bruker AVANCE 400 instrument (Bruker Biospin GmbH, Karlsruhe, Germany) operating at 400.13 MHz at 298 K. NMR samples were prepared in either CDCl_3_ or D_2_O in concentrations ranging from 10 to 15 mg/mL. Chemical shifts were reported relative to the deuterated NMR solvents: for CDCl_3_ (7.26 ppm); whereas in the case of D_2_O as a solvent, the acetone (2.22 ppm) peak was used as the internal reference. IR measurements were performed with an AVATAR 370 FT-IR instrument (Thermo Nicolet Corporation, Madison, WI, USA) with 32 scans and a resolution of 4 cm^−1^. Samples were mixed thoroughly with KBr and then pressed into pellets with a Specac compressor (Specac Inc., Smyrna, GA, USA). Equivalent quantities of reagents were calculated on the basis of per glucosamine monomer unit. The degree of substitution for acetylation (DA) of CS-(**i–v**) was estimated by following Equation (1) using ^1^H NMR spectra of the corresponding parent chitosans (**1^i–v^**).


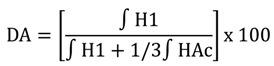
(1)

### 3.3. Gel Permeation Chromatography (GPC)

GPC analysis was used for Mw determination of chitosan, chitosan mesylate and the final quaternary alkyl or acyl derivatives of chitosan. GPC measurements were performed using the Polymer Standards Service (PSS) GmbH, Mainz, Germany, WinGPCUnichromon Dionex HPLC system equipped with a series of three columns (Novema 10 μ guard (50 × 8 mm), Novema 10 μ 30 Å (150 × 8 mm) and Novema 10 μ 1000 Å (300 × 8 mm)) (PSS GmbH, Mainz, Germany) and PSS’s ETA-2010 viscometer and Shodex RI-101 detectors (Shodex/Showa Denko Europe GmbH, Munich, Germany) using the Dionex Ultimate 3000 HPLC system (Thermo Scientific-Dionex Softron GmbH, Germering, Germany), Dionex Ultimate 3000 HPLC pump and Dionex Ultimate 3000 autosampler (Thermo Scientific-Dionex Softron GmbH, Germering, Germany). WINGPC Unity 7.4 software (PSS GmbH, Mainz, Germany) was used for data collection and processing. The eluent for the CS starting materials and all synthesized materials was 0.1 M NaCl + 0.1% TFA in Millipore water, and the standards used for the universal calibration curve were poly(2-vinylpyridine) (provided by the PSS-kit) (PSS GmbH, Mainz, Germany) with varying average molecular weight. All samples were dissolved in the same eluent as mentioned above, filtered through a 0.45-μL filter (Spartan 13/0.45 RC, Whatman GmBH, Dassel, Germany) before measurement, and the measurements were carried out at 25 °C using a flow rate of 1 mL/min and a 100 μL sample volume.

### 3.4. Chemical Synthesis

#### 3.4.1. General Procedure for *N*-Quaternized-acetyl-chitosan Derivatives

Silyl chitosan **3^i^**^–**v**^ was prepared from chitosan mesylate (Mes-CS) (**2^i^**^–**v**^) using a previously reported procedure [22]. Silyl chitosan **3^i^**^–**v**^ (2.6 mmol) was treated with Et_3_N (13 mmol) and bromoacetyl bromide (10 mmol) in dry CH_2_Cl_2_ (15 mL) under N_2_ atmosphere at −20 °C for 1 h. Concentration *in vacuo* followed by trituration with CH_3_CN afforded *N*-(bromoacetyl)-3,6-di-*O*-TBDMS-chitosan (BrA-diTBDMS-CS) (**4^i^**^–**v**^). Compound **4^i^**^–**v**^ was then treated with excess of Me_3_N or pyridine to afford *N*-(2-(*N*,*N*,*N*-trimethylammoniumyl)acetyl)-3,6-di-*O*-TBDMS-chitosan bromide (**5^i^**^–**v**^) and *N*-(2-(*1*-pyridiniumyl)acetyl)-3,6-di-*O*-TBDMS-chitosan bromide (**7^i^**^–**v**^), respectively.

#### 3.4.2. General Procedure for *N*-Quaternized-hexanoyl-chitosan Derivatives

Silyl chitosan **3^i^**^–**v**^ (1.3 mmol) was treated with Et_3_N (6.5 mmol) and 6-bromohexanoyl chloride (5.2 mmol) in dry CH_2_Cl_2_ (15 mL) under N_2_ atmosphere at −20 °C for 1 h. Concentration *in vacuo* followed by trituration with CH_3_CN afforded *N*-(6-bromohexanoyl)-3,6-di-*O*-TBDMS-chitosan (BrHA-diTBDMS-CS) (**9^i^**^–**v**^). Compound **9^i^**^–**v**^ was then treated with an excess of either Me_3_N or pyridine to afford *N*-(6-(*N*,*N*,*N*-trimethylammoniumyl)hexanoyl)-3,6-di-*O*-TBDMS-chitosan bromide/iodide (**10^i^**^–**v**^) and *N*-(6-(*1*-pyridiniumyl)hexanoyl)-3,6-di-*O*-TBDMS-chitosan bromide/iodide (**12^i^**^–**v**^), respectively.

#### 3.4.3. General TBDMS Deprotection Procedure to Give the Final Quaternary Ammoniumyl and Pyridiniumyl Derivatives (**6^i^**^–**v**^, **8^i^**^–**v**^, **11^i^**^–**v**^, **13^i^**^–**v**^)

The compounds (**5^i^**^–**v**^, **7^i^**^–**v**^, **10^i^**^–**v**^ or **12^i^**^–**v**^) were stirred in MeOH (4–5 mL) and conc HCl (1–2 mL) for 12 h at 25 °C. Purification was done by dialysis followed by freeze-drying to afford the corresponding quaternized product (**6^i^**^–**v**^, **8^i^**^–**v**^, **11^i^**^–**v**^ or **13^i^**^–**v**^).

#### 3.4.4. General Procedure for *N*-Quaternized-chitosan

Silyl chitosan **3^i^**^–**v**^ (3.6 mmol) was treated with cesium carbonate (Cs_2_CO_3_) (14.2 mmol) and CH_3_I (17.8 mmol) in dry NMP (20 mL) at 50 °C for 48 h. Precipitation in ice-cold water followed by filtration afforded *N*,*N*,*N*-trimethyl-3,6-di-*O*-TBDMS-chitosan iodide (**14^i^**^–**v**^). Compound **14^i^**^–**v**^ (3.30 mmol) was then deprotected by treatment with tetrabutyl ammonium fluoride (TBAF) (1 molar) solution in NMP (10 mL) at 50 °C for 48 h. Purification was done by dialysis followed by freeze-drying to afford *N*,*N*,*N-*trimethyl chitosan chloride (TMC) (**15^i^**^–**v**^).

Note: details of all experimental procedures with assignments of ^1^H NMR spectra are available in the Supplementary Information.

### 3.5. Biological Methods

#### 3.5.1. Bacterial Strains, Media and Culture Conditions

The antibacterial tests were assayed according to standard Clinical and Laboratory Standards Institute (CLSI) methods for antimicrobial dilution susceptibility tests [54]. Minimum inhibitory concentration (MIC) and minimum lethal concentration (MLC) values were measured against *Staphylococcus aureus* (*S. aureus*, ATCC 29213) and *Escherichia coli (E. coli*, ATCC 25922) obtained from the American Type Culture Collection, representing Gram positive and Gram negative bacteria that are susceptible to routinely measured antibiotics. The broth microdilution method was used to determine the MIC values using Mueller-Hinton Broth (Oxoid, Hampshire, UK) at pH 7.2 as the medium. Blood agar (heart infusion agar (Oxoid) with 5% (v/v) defibrinated horse blood) was used for the measurement of MLC. The samples were prepared by dissolving chitosan derivatives in sterile water to an initial concentration of 32,768 μg/mL. Fifty microliters of each sample were added to the first two wells on a micro-titer plate, and two-fold dilutions were done in 50 μL of Mueller-Hinton broth from well two on. This gave a final range varying from 16,384 μg/mL to 16 μg/mL, with the option of reporting 32,768 μg/mL as the highest concentration. Gentamicin was used as the positive control during the test. Bacterial solution of 0.5 McFarland suspension (1–2 × 10^8^ CFU/mL) was prepared by direct colony suspension in Mueller-Hinton broth and further diluted 100-fold so as to achieve a final test concentration of bacteria of approximately 1 × 10^6^ CFU/mL (or 5 × 10^5^ CFU/well in the microtiter plate). The microtiter plates were then incubated at 35 °C for 18 h under moistened conditions. The MIC value was defined as the lowest concentration of the antibacterial agent that completely inhibited visible growth of the microorganism in the microtiter plate. For MLC measurement, 10 μL × 2 of each of the dilutions that showed no visible growth were plated on a blood agar plate and incubated at 35 °C for 18 h. MLC was defined as the lowest concentration that achieved a 99.9% decrease in viable cells.

#### 3.5.2. Hemolytic Activity

Hemolysis assays were performed according to previously published procedures [55,56] with slight modification. Human red blood cell concentrate having an RBC of 6.45 × 10^12^/L, total hemoglobin of 201 g/L and WBC of 0.15 × 10^9^/L was used for testing the hemolytic activity of the CS derivatives. RBCs (100 μL) were suspended in 10 mL of TBS (pH = 7.2). The polymer solutions were prepared in TBS at an initial concentration of 32,768 μg/mL and serially diluted 2-fold in a 96-well plate, so as to have a minimum concentration of 16 μg/ mL. One hundred microliters of RBC suspension were added to 100 μL of the polymer solutions and incubated at 37 °C with light shaking for 30 min. Cells treated with TBS and 1% Triton-X100 were used as negative and positive controls, respectively. The cell suspensions were centrifuged at 1500 rpm for 10 min, and the supernatant was collected to measure the absorbance of the released hemoglobin at 540 nm on a Thermo Scientific Multiscan Spectrum Photometer. The percentage hemolysis was calculated using the following equation:

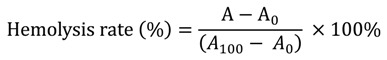
(2)
where A = absorbance of the polymer solutions, A_0_ = absorbance of negative control and A_100_ = absorbance of positive control.

#### 3.5.3. Cytotoxicity

The cytotoxicity of the compounds were determined on the colorectal adenocarcinoma-derived cell line, Caco-2 (ATCC). Cells were grown in EMEM medium (ATCC) supplemented with l-glutamine, sodium pyruvate, 10% FBS (Invitrogen) and streptomycin/penicillin. Cells were grown until 80% confluent at 37 °C, in 5% CO_2_ and then seeded at 1000 cells/well in a 96-well plate. After 48 h to allow for attachment and initial proliferation of the cells, the compounds were added at the specified concentrations in full culture media for 24 h. As a positive control, 1% Triton-X100 in PBS was used. After the incubation period, the cell culture medium containing the compounds was removed to prevent media color changes from influencing the spectroscopic measurements. Fresh cell culture medium was added and the cells were left to equilibrate for 30 min. Cytotoxicity was determined using the XTT assay (ATCC) following the manufacturer’s instructions. The XTT reagent was incubated for 3 h at 37 °C in the dark before measuring optical density in a Multi Skan spectrometer (Thermo Scientific, Waltham, MA, USA) at 475 nm and 660 nm to account for non-specific absorbance. The compounds were tested in triplicate at each concentration, and the results in each case are presented as the half maximal effective concentration (EC_50_).

## 4. Conclusions

There has been significant interest in the study of antimicrobial chitosan derivatives, as is evident from the many recent original publications [34,57,58,59] and reviews [27,60,61]. However, taken together, previous studies have not provided a clear picture of the structure-activity relationship. This is possibly due to a lack of uniformity in the products synthesized and also due to insufficient characterization. Reactions performed directly on chitosan are non-selective and usually give rise to heterogeneous products.

In the current study, we used a systematic approach to investigate how certain chemical characteristics can affect the biological activity of chitosan derivatives. The results were validated by synthesizing each type of derivative from five different starting materials. Reproducible synthesis is important for this kind of investigation and, in this case, the series of cationic chitosan derivatives was synthesized using a TBDMS protection strategy (diTBDMS-CS) under homogeneous conditions. This provided full substitution on free amino groups. Two functional groups, trimethylammonium and the pyridinium group, were successfully inserted into the polymer either directly or with the help of C-2 and C-6 spacers and could be well characterized by spectroscopic techniques. The MIC values for the compounds revealed that the inhibitory effect was higher against *S. aureus*, as compared to *E. coli*. The MLC values were at most one dilution higher than the MIC values for most of the compounds, showing that many of the compounds were bactericidal within the measured concentration range. Furthermore, the derivatives with trimethylammonium as the quaternary group showed higher activity than the derivatives with the pyridinium group. Amongst all of the derivatives, TMC showed the highest antibacterial activity with MIC values as low as 4 μg/mL. On the other hand, a descending order in activity could be observed with increasing spacer length in the compounds from C-2 to C-6. TMC displayed moderate hemolytic activity, which decreased with a decrease in DA values, while all of the other derivatives remained non-hemolytic within the measured concentration range. Hence, a decrease in activity against RBC was observed with increasing spacer length. A decrease in cytotoxicity with decreasing DA and increasing Mw was also observed in most cases, while no particular trend in cytotoxicity with changes in chain length could be derived. In spite of their cellular cytotoxicity, these highly active TMC, TMAC and PyAC derivatives should be considered as antibacterial agents for topical applications and disinfecting medical equipment. Thus, these results have increased our understanding of the effect of the positioning of the cationic charge, as well as the effects of DA, DS and Mw (for relatively low Mw chitosan derivatives) on the antibacterial properties of this class of agent.

## Supplementary Files

Supplementary File 1
