# Supplementary material for: The Effect of Substituent, Degree of Acetylation and Positioning of the Cationic Charge on the Antibacterial Activity of Quaternary Chitosan Derivatives
Source: Mar Drugs. 2014 Aug 21;12(8):4635–58. doi: 10.3390/md12084635 (PMC4145335; doi:10.3390/md12084635)
Supplement: Supplementary File 1 [file marinedrugs-12-04635-s001.pdf]

Supplementary Information

Table of Contents

1. Synthetic Procedure Page S2–S3
2. General Procedures for *N*-Quaternized-hexanoylchitoderivatives Page S3–S5
3. General Procedure for *N*-Quaternizedchitosan Page S5
4. Figures S1–S4. ^{1}H NMR spectra of key chitosan intermediates compounds Page S6–S7
5. Figures S5–S32. ^{1}HNMR spectra of final chitosan derivatives Page S8–S21
6. Graphs for Hemolysis Rate Page S22–S24

1. Synthetic Procedure

1.1. Chitosan Mesylate (Mes-CS) (2$^{i–v}$)

Free base chitosan (**1**$^{i–v}$) (1 g, 6 mmol) was suspended in methanesulfonic acid (CH_3SO_3H) (10 mL, 0.153 mol) and cooled to 10 °C. To the reaction mixture, H_2O (~10 mL) was added dropwise until a clear homogeneous solution was obtained. The reaction mixture was then stirred for 1 h before precipitating with EtOH (40 mL) resulting in a gel-like precipitate. The precipitate was filtered under suction using a sintered funnel and washed with EtOH (3 × 25 mL), followed by washing with acetone (3 × 20 mL). The material was then allowed to air-dry for 1 h. This salt precipitate was redissolved in a minimum quantity of H_2O (5–10 mL) and reprecipitated using acetone (60 mL), filtered, washed with acetone (2 × 30 mL) and the obtained material further dried in a vacuum oven at 40 °C overnight to afford corresponding finely powdered off-white chitosan mesylate salt (**2**$^{i–v}$) (1.39 g, 90%). FT-IR (KBr): ν 3439 (O–H), 2935 (C–H), 1636 (C=O amide I), 1526 (C=O amide II), 1384 (C–H), 1198–1059 (C–C, C–O) cm^{-1}. ^{1}H NMR (400 MHz, D_2O): δ 2.06 (s, CH_3C=O (Glc-NAc)), 2.81 (s, CH_3S), 3.17 (m, H-2 (GlcN)), 3.6–4.1 (m, H-2 (GlcNAc), H-3–H-6′), 4.86 (H-1, partially overlapped with the HOD peak) ppm.

1.2. 3,6-di-O-tert-Butyldimethylsilyl-chitosan (diTBDMS-CS) (3$^{i–v}$)

Chitosan mesylate (**2**$^{i–v}$) (1 g, 3.97 mmol) was dissolved in dry DMSO (15 mL) under N_2 atmosphere. To this reaction mixture, imidazole (2.71 g, 39.74 mmol) and TBDMSCl (2.99 g, 19.87 mmol) in dry DMSO (13 mL) wereadded dropwise, and the resulting mixture was stirred at 25 °C. During the addition of the reagents, the reaction mixture turned cloudy, and eventually, sometime after completion of the addition, a solid gel-type material separated out from the solution. The reaction mixture was stirred for 24 h at 25 °C and then filtered by using a sintered funnel, and the solid obtained was continuously triturated while washing with H_2O (5 × 30 mL), followed by washing with CH_3CN (3 × 20 mL). The material was air dried and then further dried in a vacuum oven at 40 °C overnight, to afford corresponding off-white finely powdered silyl protected DiTBDMS-CS compound (**3**$^{i–v}$) (1.46 g, 96%).FT-IR (KBr): ν 3403 (N–H), 2957–2858 (C–H), 1668 (C=O amide I), 1568 (C=O amide II), 1474 (C–H), 1390–1362 (C–H), 1258 (Si–CH_3, C–N), 1109–1050 (C–O, Si–O), 836–777 (Si-C) cm^{-1}. ^{1}H NMR (400 MHz, $CDCl_3$): δ 0.04–0.12 (br s, 12H, $(CH_3)_2Si$), 0.89–0.90 (br s, 18H, $(CH_3)_3C$), 1.99 (br s, CH_3C=O (GluNAc)), 2.71 (br s, H-2), 3.32 (br s, H-5), 3.49 (br s, H-3), 3.67 (br s, H-4), 3.85–3.90 (br s, H-6, H-6′), 4.29 (br s, H-1) ppm.

1.3. N-(Bromoacetyl)-3,6-di-O-TBDMS-chitosan (BrA-diTBDMS-CS) (4$^{i–v}$)

Silyl chitosan **3**$^{i–v}$ (1 g, 2.6 mmol) was dissolved in dry CH_2Cl_2 (15 mL) under N_2 atmosphere. The reaction mixture was cooled to −20 °C by using a salt-ice cooling mixture. To the reaction mixture, Et_3N (1.81 mL, 13 mmol) was added, followed by the slow dropwise addition of bromoacetyl bromide (0.91 mL, 10 mmol). Stirring was continued for 1 h at a constant temperature maintained at −20 °C before the reaction mixture was diluted with CH_2Cl_2 (30 mL) and concentrated *in vacuo*. The crude material obtained was triturated and stirred in CH_3CN (15 mL), filtered and washed with CH_3CN

(3 × 15 mL) and air-dried. Dry material was redissolved in CH_2Cl_2 (45 mL) and washed with H_2O (3 × 30 mL) and brine (20 mL). The organic layer was dried over anhydrous Na_2SO_4 and concentrated *in vacuo* to afford corresponding pale yellow powdered bromoacetyl intermediate $\mathbf{4}^{i–v}$ (1.2 g, 92%). FT-IR (KBr): ν 3401 (N–H), 2957–2858 (C–H), 1682 (C=O amide I), 1530 (C=O amide II), 1473 (C–H), 1391–1362 (C-H), 1259 (Si–CH_3, C–N), 1101–1050 (C–O, Si–O), 837–778 (Si–C) cm^{-1}. ^1H NMR (400 MHz, $CDCl_3$): δ 0.07–0.14 (br s, 12H, $(CH_3)_2Si$), 0.88–0.89 (br s, 18H, $(CH_3)_3C$), 2.0 (br s, CH_3C=O (GluNAc)), 3.25–4.02 (m, H-2–H-6′ and –CH_2Br), 4.43 (br s, H-1) ppm.

1.4. N-(2-(N,N,N-Trimethylammoniumyl)acetyl)-3,6-di-O-TBDMS-chitosan Bromide ($\mathbf{5}^{i–v}$)

Freshly prepared bromoacyl intermediate $\mathbf{4}^{i–v}$ (450 mg, 0.9 mmol) was dissolved in CH_2Cl_2 (10 mL) under N_2 atmosphere. Excess Me_3N (4.2 molar in EtOH) (10 mL) was added, and the resulting mixture was stirred for 24 h at 25 °C. The reaction mixture was concentrated *in vacuo* to isolate the corresponding crude Product $\mathbf{5}^{i–v}$. These crude materials were used directly for the next deprotection step without any purification and characterization.

1.5. N-(2-(1-Pyridiniumyl)acetyl)-3,6-di-O-TBDMS-chitosan Bromide ($\mathbf{7}^{i–v}$)

Freshly prepared bromoacyl intermediate $\mathbf{4}^{i–v}$ (400 mg, 0.8 mmol) was dissolved in CH_2Cl_2 (5 mL) and stirred in excess pyridine (15 mL) for 24 h at 25 °C under N_2 atmosphere. The reaction mixture was concentrated completely *in vacuo* to isolate the corresponding crude product ($\mathbf{7}^{i–v}$). These crude materials were used directly for the next deprotection stage without any further purification or characterization.

2. General Procedures for *N*-Quaternized-hexanoyl-chitosan Derivatives

2.1. N-(6-Bromohexanoyl)-3,6-di-O-TBDMS-chitosan (BrHA-diTBDMS-CS) ($\mathbf{9}^{i-v}$)

Silyl chitosan $\mathbf{3}^{i–v}$ (0.5 g, 1.3 mmol) was dissolved in dry CH_2Cl_2 (15 mL) under N_2 atmosphere. The reaction mixture was cooled to –20 °C by using a salt-ice mixture. To the reaction mixture, Et_3N (0.91 mL, 6.5 mmol) was added followed by the slow dropwise addition of 6-bromohexanoyl chloride (0.8 mL, 5.2 mmol). The stirring continued for 1 h while constantly maintaining a temperature at −20 °C. The reaction mixture was diluted with CH_2Cl_2 (35 mL) and concentrated *in vacuo*. The isolated crude product was triturated and stirred with CH_3CN (30 mL), filtered and the solid obtained washed with fresh CH_3CN (3 × 20 mL) before air-drying to afford the bromohexanoyl intermediate ($\mathbf{9}^{i–v}$) as a white solid (504 mg, 68.5%). FT-IR (KBr): ν 3342 (N–H), 2956–2858 (C–H), 1677 (C=O amide I), 1527 (C=O amide II), 1473 (C–H), 1390–1362 (C–H), 1257 (Si–CH_3, C–N), 1096–1054 (C–O, Si–O), 838–778 (Si–C) cm^{-1}. ^1H NMR (400 MHz, $CDCl_3$): δ 0.06–0.12 (br s, 12 H, $(CH_3)_2Si$), 0.88–0.89 (br s, 18H, $(CH_3)_3C$), 1.46 (br m, 2H,–CH_2– [a]), 1.66 (br m, 2H, –CH_2–[a]), 1.87 (br m, 2H, –CH_2–[a]), 2.0 (br s, CH_3C=O (GluNAc)), 2.23 (br m, 2H, $COCH_2$–), 3.39 (br s, 2H,–CH_2Br), 3.56–4.32 (br m, 7H, H-1–H-6′) ppm. ([a]Please refer to Figure 4C)

*2.2. N-(6-(N,N,N-Trimethylammoniumyl)hexanoyl)-3,6-di-O-TBDMS-chitosan Bromide/Iodide (**10**$^{i–v}$)*

Freshly prepared bromohexanoyl compound (**9**$^{i–v}$) (450 mg, 0.8 mmol) was dissolved in CH_2Cl_2 (12 mL) under N_2 atmosphere. To the reaction mixture, excess Me_3N (4.2 molar in EtOH) (15 mL) was added, and the resulting mixture was stirred for 24 h at 25 °C. Some white precipitate was formed as the reaction progressed, and this increased after the addition of a catalytic amount of KI. The stirring was continued further for 24 h. The reaction mixture was concentrated *in vacuo* to isolate the corresponding crude Product **10**$^{i–v}$. These crude materials were washed with diethyl ether, filtered, dried and then used directly for the next deprotection stage without any further purification or characterization.

*2.3. N-(6-(1-Pyridiniumyl)hexanoyl)-3,6-di-O-TBDMS-chitosan Bromide/Iodide (**12**$^{i–v}$)*

Freshly prepared bromohexanoyl compound (**9**$^{i–v}$) (400 mg, 0.71 mmol) was dissolved in CH_2Cl_2 (5 mL) under N_2 atmosphere. To the reaction mixture, excess quantity of pyridine (15 mL) and catalytic amount of KI was added, and the resulting mixture continued to be stirred for 48 h at 25 °C. The reaction mixture was concentrated completely *in vacuo* to isolate the corresponding crude product (**12**$^{i–v}$). These crude materials were washed with diethyl ether, filtered, dried and then used directly for the next deprotection stage without any purification and characterization.

*2.4. General TBDMS Deprotection Procedure to Give the Final Quaternary AmmoniumylandPyridiniumylDerivatives(**6**$^{i–v}$, **8**$^{i–v}$, **11**$^{i–v}$, **13**$^{i–v}$)*

The compounds (**5**$^{i–v}$, **7**$^{i–v}$, **10**$^{i–v}$ or **12**$^{i–v}$) (300 mg)weredissolved in MeOH (4–5 mL), and concHCl (1–2 mL) was added. The reaction mixture was stirred for 12 h at 25 °C, diluted with H_2O (10 mL) and, then, ion exchanged by adding 10% NaCl (aqueous) (w/v) (15 mL), and the resulting mixture was stirred for 1 h at 25 °C. The colorless solution was then dialyzed against 5% aqueous NaCl solution for oneday and then against deionized water for twodays, before it was freeze-dried to afford the corresponding deprotectedwater-soluble, white, fluffy quaternized product (**6**$^{i–v}$, **8**$^{i–v}$, **11**$^{i–v}$ or **13**$^{i–v}$). The deprotection was repeated if needed to remove traces of silyl impurities that could be observed in ^{1}H NMR after the first round.

N-(2-(*N*,*N*,*N*-trimethylammoniumyl)acetyl)-chitosan chloride (TMA-CS) (**6**$^{i–v}$). FT-IR (KBr): ν 3419 (O–H, N–H), 2956 (C–H), 1684 (C=O amide I), 1568 (C=O amide II), 1476 (C–H), 1067 (C–O) cm^{-1}. ^{1}H NMR (400 MHz, D_2O): δ 2.05 (br s, CH_3C =O (GluNAc), 3.32 (br s, $^+N(CH_3)_3$), 3.5–3.9 (m, H-2–H-6′), 4.17 (br s, CH_2C=O), 4.63 (br s, H-1) ppm. *N*-(2-(*1*-pyridiniumyl)acetyl)-chitosan chloride (PyA-CS) (**8**$^{i–v}$). FT-IR (KBr): ν 3400 (O–H, N–H), 3060, 2930 (C–H), 1685 (C=O amide I), 1636 (C=C, aryl subst.), 1567 (C=O amide II), 1490 (C=C–C, aromatic ring), 1363–1228 (C–N, aryl), 1071 (C–O) cm^{-1}. ^{1}H NMR (400 MHz, D_2O): δ 2.06 (br s, CH_3C=O (GluNAc), 3.5–4.0 (m, H-2–H-6′ and Py^+CH_2C=O), 4.71 (br s, H-1, partially overlapped with HOD peak), 8.16 (br t, *J* = 8 Hz, 2H, Py^+*m*-CH), 8.67 (br t, *J* = 8 Hz, 1H, Py^+*p*-CH), 8.82 (br d, *J* = 8 Hz, 1H, Py^+*o*-CH) ppm. *N*-(6-(*N*,*N*,*N*-trimethylammoniumyl)hexanoyl)-chitosan chloride (**11**$^{i–v}$). FT-IR (KBr): ν 3424 (O–H, N–H), 2950–2871 (C–H), 1652 (C=O amide I), 1555 (C=O amide II), 1481 (C–H), 1069 (C–O) cm^{-1}. ^{1}H NMR (400 MHz, D_2O): δ1.41 (br p, 2H,–CH_2– ^{a}), 1.68 (br p, 2H, –CH_2–^{a}), 2.07 (br s, CH_3C=O (GluNAc)), 2.36 (br q, 2H, –COCH_2–^{a}), 3.12 (br s, $^+N(CH_3)_3$),

3.32 (brt, 2H, –$CH_2NMe_3^{+a}$), 3.5–4.0 (br m, H-2–H-6′), 4.58 (br s, H-1) ppm. ([a]See Figure 4D). *N*-(6-(*1*-pyridiniumyl)hexanoyl)-chitosan chloride (**13**$^{\mathbf{i–v}}$). FT-IR (KBr): ν 3423 (O–H, N–H), 3062, 2936–2867 (C–H), 1651 (C=O amide I), 1556 (C=O amide II), 1488 (C=C–C, aromatic ring), 1374–1316 (C–N, aryl), 1068 (C–O) cm^{-1}.^{1}H NMR (400 MHz, D_2O): δ 1.39 (br p, 2H,–CH_2–[a]), 1.65 (br m, 2H,–CH_2–[a]), 2.06 (br p, 2H (–CH_2– [a]) overlapped with CH_3C=O (GluNAc)), 2.30 (br m, 2H, –$COCH_2$–[a]), 3.4–4.0(br m, H-2–H-6′), 4.55 (br s, H-1), 4.61 (br t, 2H, –CH_2Py^{+a}), 8.09 (br t, *J* = 8 Hz, 2H, Py^+*m*-CH), 8.57 (br t, *J* = 8 Hz, 1H, Py^+*p*-CH), 8.84 (br d, *J* = 8 Hz, 1H, Py^+*o*-CH) ppm. ([a]See Figure 4E).

3. General Procedure for *N*-Quaternized-chitosan

*3.1. N,N,N-Trimethyl-3,6-di-O-TBDMS-chitosan Iodide (**14**$^{i–v}$)*

Silyl chitosan **3**$^{\mathbf{i–v}}$ (1.42 g, 3.6 mmol) was dissolved in dry NMP (20 mL). To the reaction mixture, cesium carbonate (Cs_2CO_3) (4.63 g, 14.2 mmol) was added and the solution stirred for 1 h, followed by dropwise addition of CH_3I (1.11 mL, 17.8 mmol) under cooling. The reaction was carried out in a closed reaction vial at 50 °C for 48 h. The solution was then dialyzed against deionized water for twodays and freeze-dried to afford a dark red product (**14**$^{\mathbf{i–v}}$) (1.85 g, 93%). ^{1}H NMR (400 MHz, $CDCl_3$): δ 0.01–0.31 ($(CH_3)_2Si$), 0.86–0.90 ($(CH_3)_3C$), 3.64 ($^+N(CH_3)_3$) partially overlapped by H-1 to H-6′) ppm.

*3.2. N,N,N-Trimethyl Chitosan Chloride (TMC) (**15**$^{i–v}$)*

Compound**14**$^{\mathbf{i–v}}$ (1.85 g, 3.30 mmol) was deprotected by treatment with tetrabutyl ammonium fluoride (TBAF) (1 molar) solution in NMP (10 mL) at 50 °C for 48 h. The resulting solution was dialyzed for two days against deionized water, then ion-exchanged with 10% NaCl (aqueous) (w/v) overnight, and this was then followed by dialysis against deionized water for another two days. The resulting compound was then freeze-dried, giving a light brown and fluffy trimethylated CS (**15**$^{\mathbf{i–v}}$) (650 mg, 74.2%). In cases where ^{1}H NMR analysis showed that the trimethylated chitosan was not fully deprotected, the deprotection process was repeated. FT-IR (KBr): ν 3422 (O–H), 2923 (C–H), 1653 (C=O amide I), 1488 (C–H), 1051 (C–O) cm^{-1}. ^{1}H NMR (400 MHz, D_2O): δ 2.08 (CH_3C=O, GlcNAc), 3.35 ($(^+N(CH_3)_3$), 3.75 (H-2), 3.90 (H-6), 3.99 (H-5), 4.36 (H-4), 4.47 (H-3), 5.49 (H-1) ppm.

4. Figures S1–S4: ^{1}H NMR Spectra of Key Chitosan Intermediates Compounds

Figure S1. Representative ^{1}H NMR (400 MHz, D_2O) spectrum_of compound **2^{iv}** (Mes-CS^{iv}).

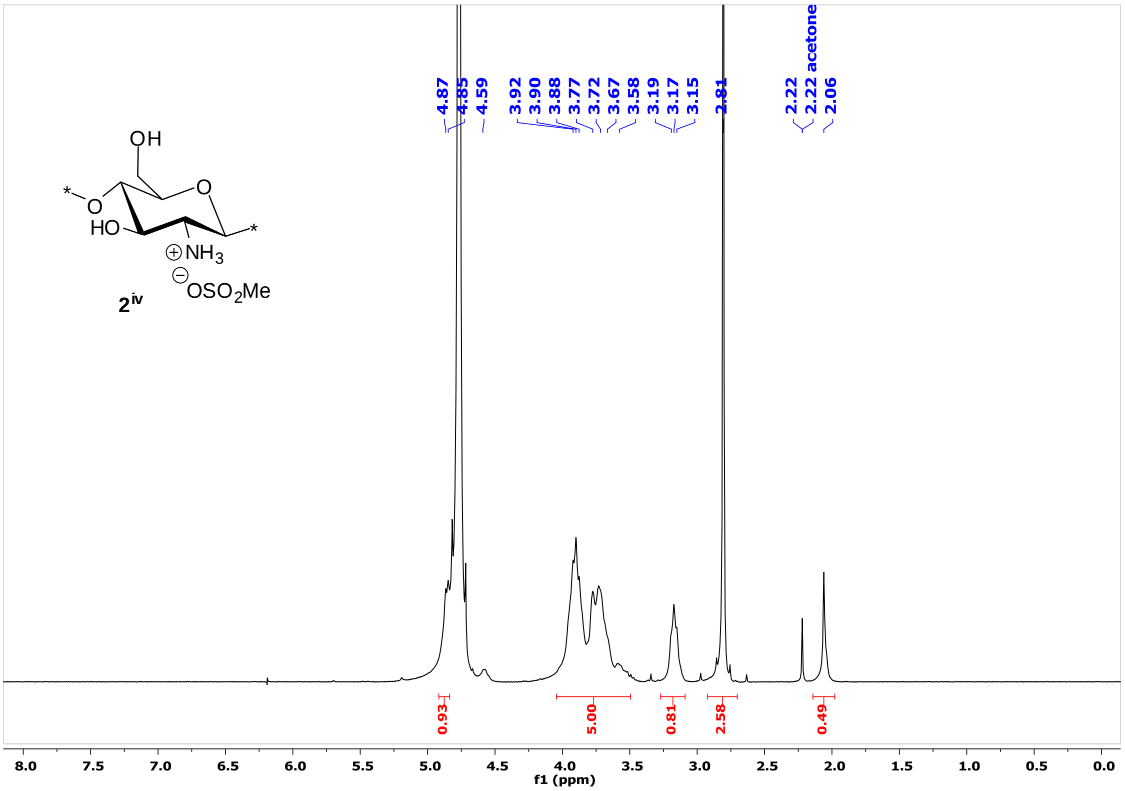

Figure S2. Representative^{1}H NMR (400 MHz, $CDCl_3$) spectrumof compound **3^{iv}** (diTBDMS-CS^{iv}).

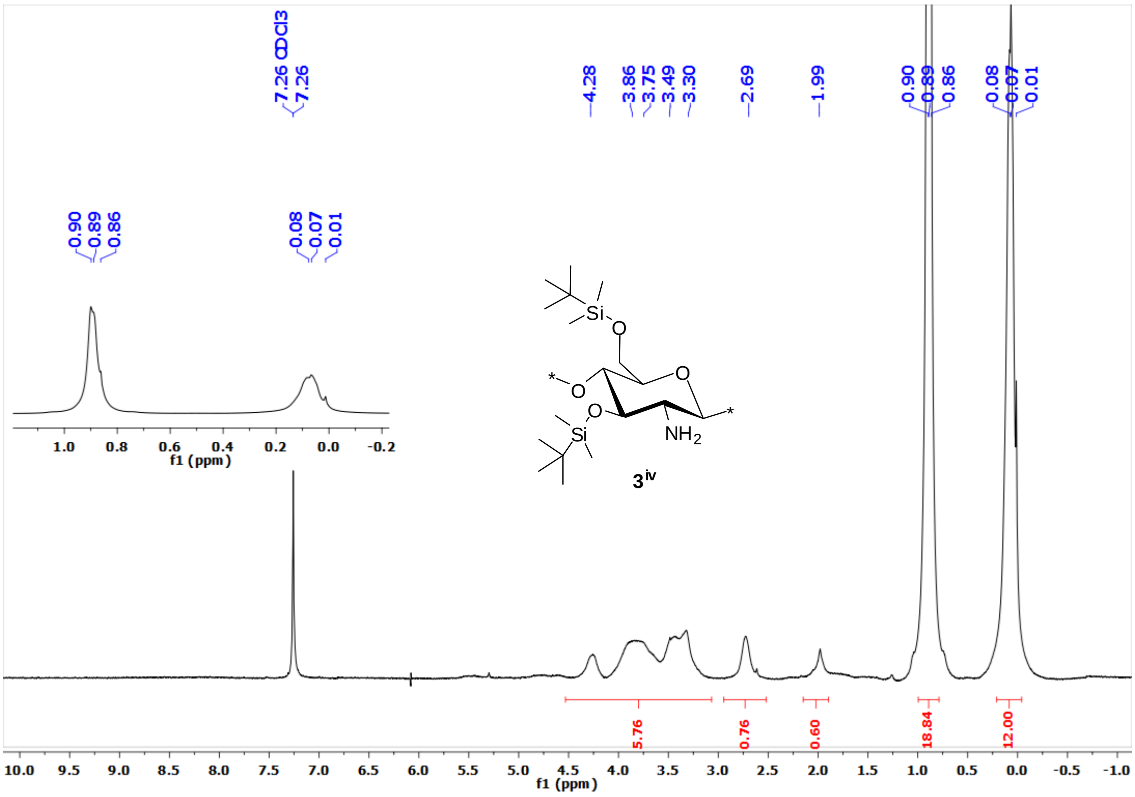

Figure S3. Representative ^{1}H NMR (400 MHz, $CDCl_3$) spectrum_of compound **4**^{i} (BrA-diTBDMS-CS^{i}).

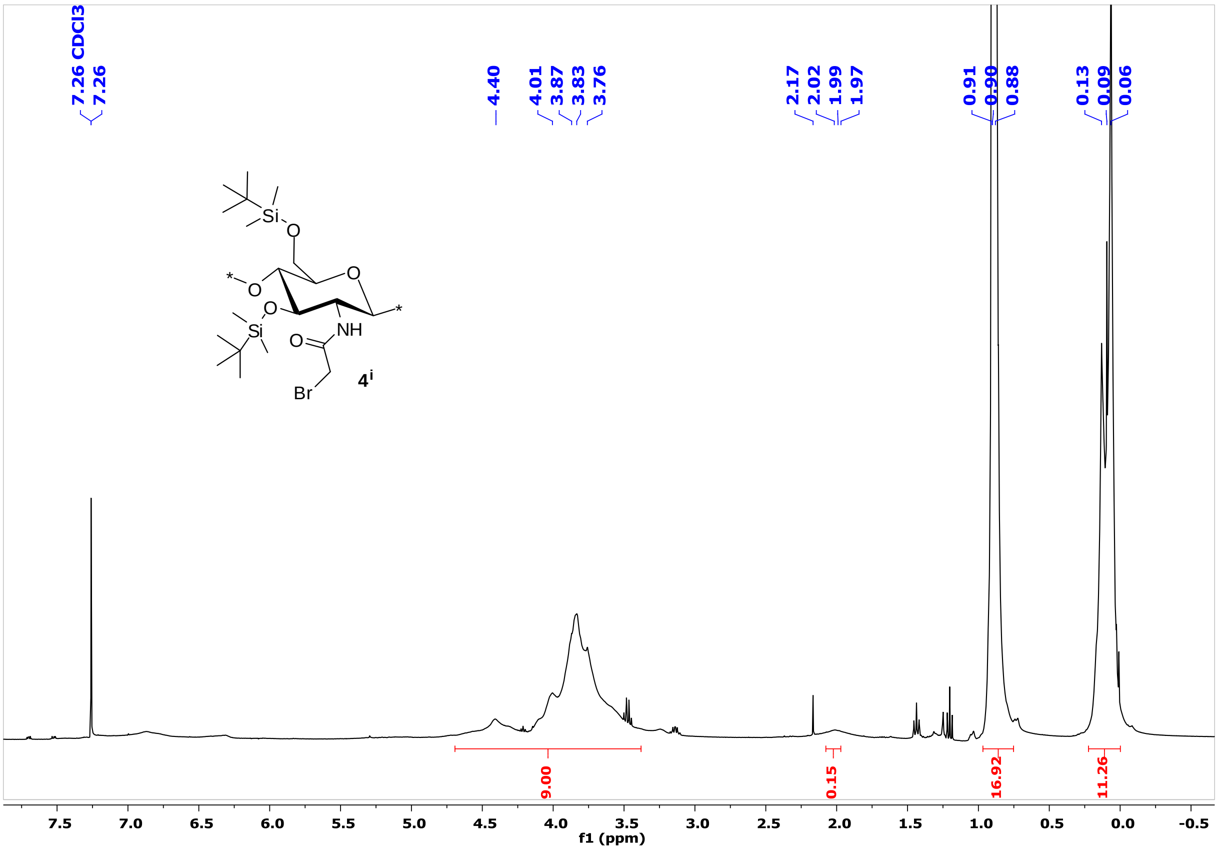

Figure S4. Representative^{1}H NMR (400 MHz, $CDCl_3$) spectrumof the key intermediate compound **9**^{iv} (BrHA-diTBDMS-CS^{iv}).

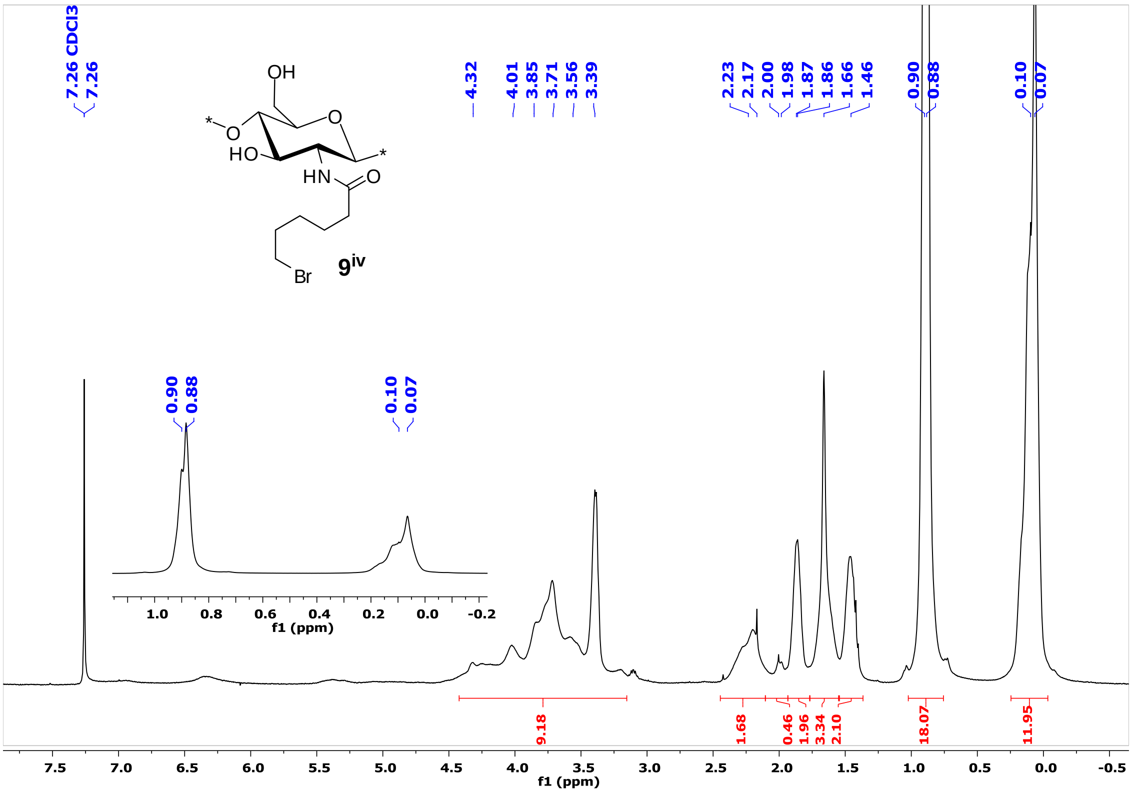

5. Figures S5–S32: ^{1}HNMR Spectra of Final Chitosan Derivatives

Figure S5. ^{1}H NMR (400 MHz, D_2O) spectrum of compound **6^i** (TMA-CS^i).

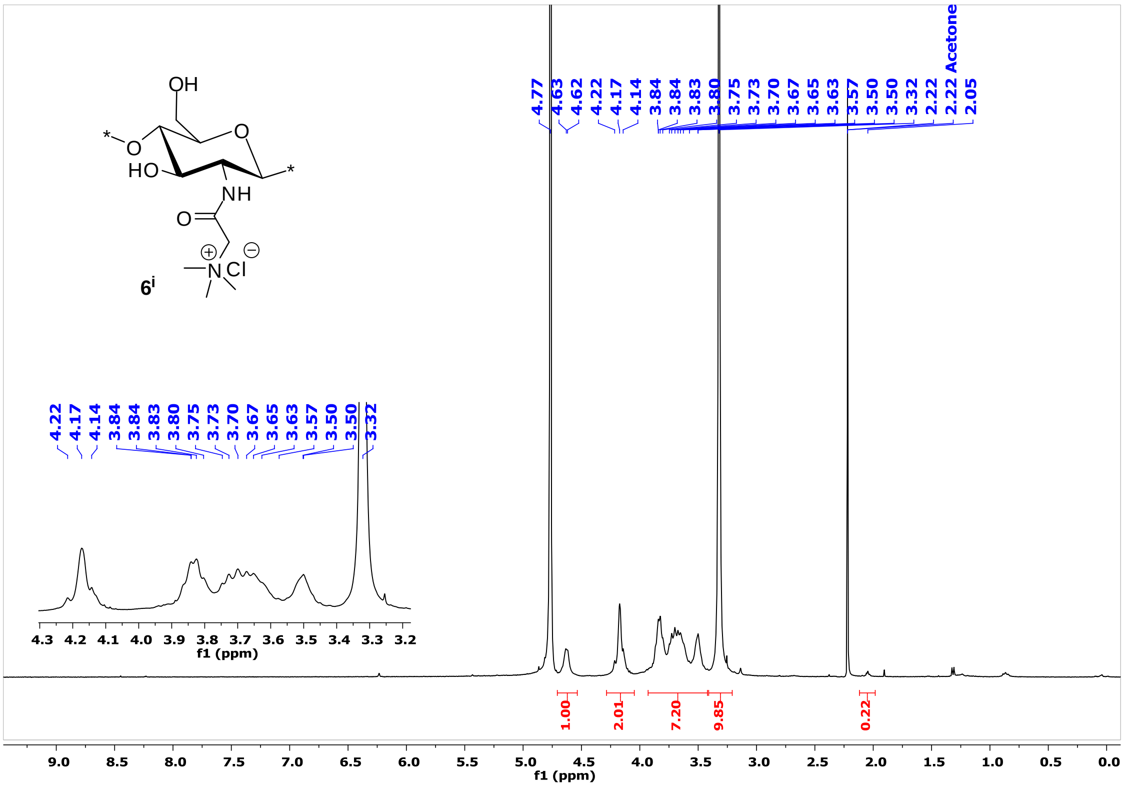

Figure S6. ^{1}H-^{1}H COSY NMR (400 MHz, D_2O) spectrum of_compound **6^i** (TMA-CS^i).

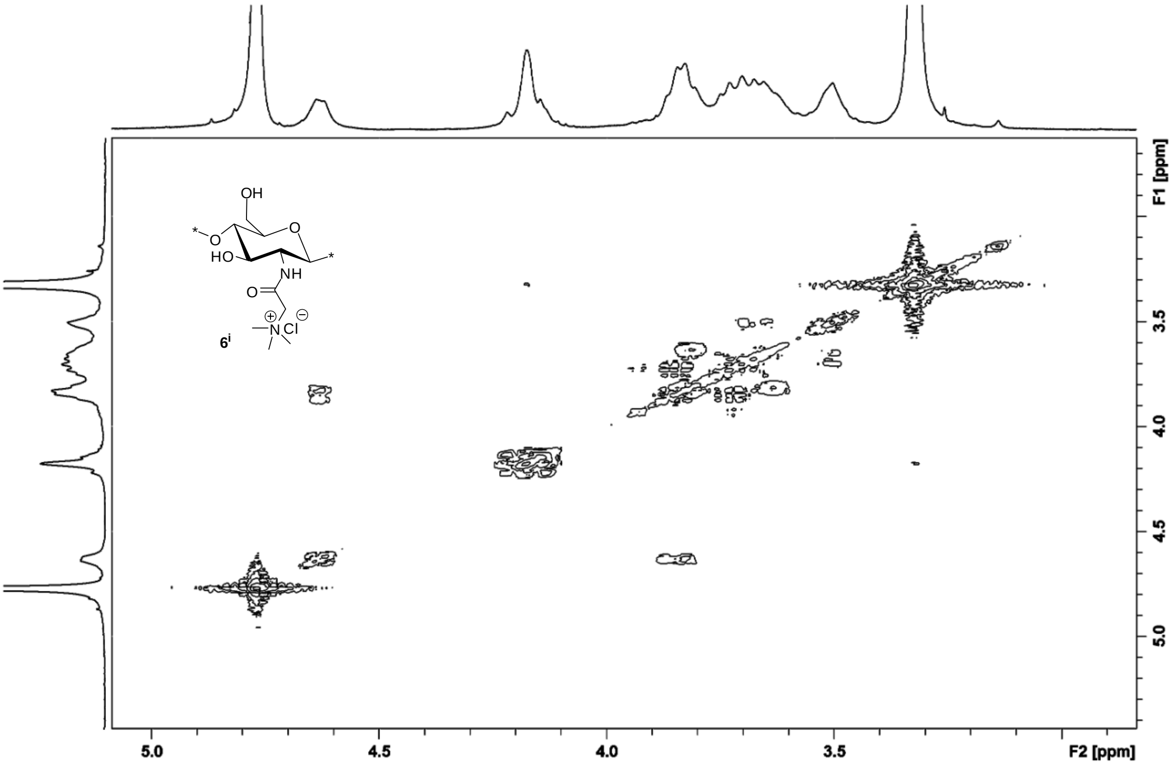

Figure S7. ^{1}H NMR (400 MHz, D_2O) spectrum of_compound **8^{i}** (PyA-CS^{i}).

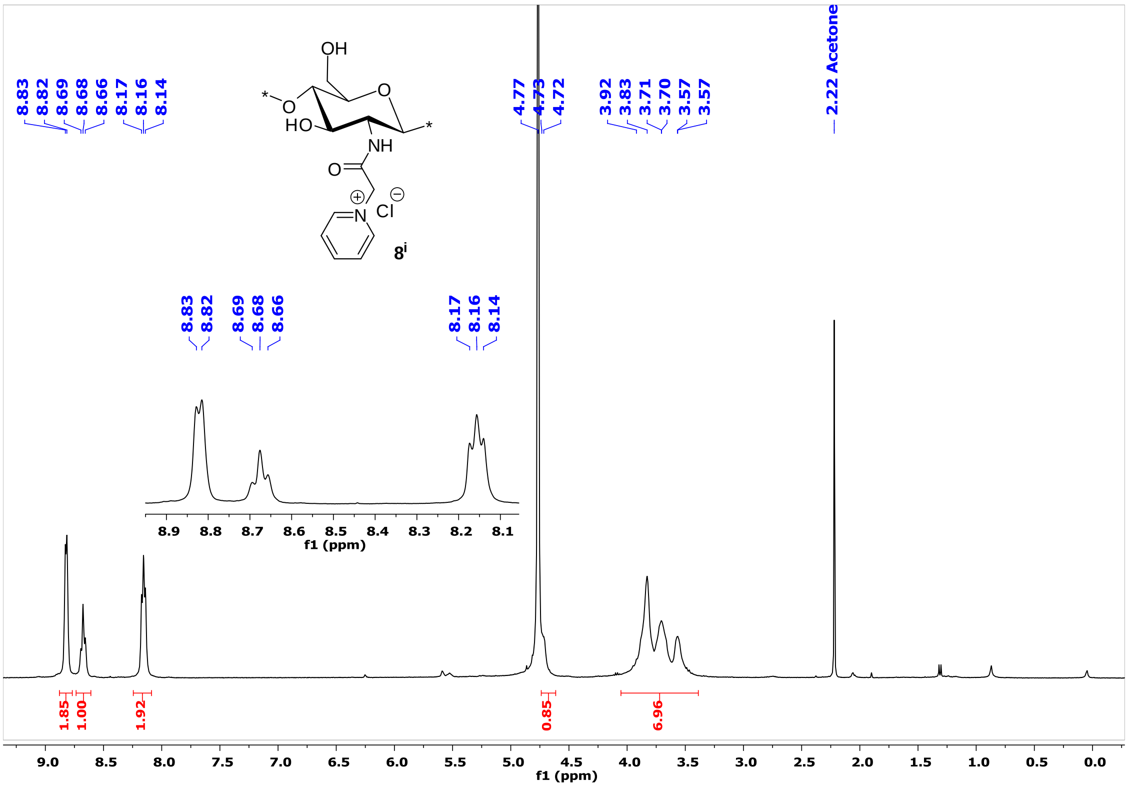

Figure S8. ^{1}H-^{1}H COSY (400 MHz, D_2O) spectrum of compound **8^{i}** (PyA-CS^{i}).

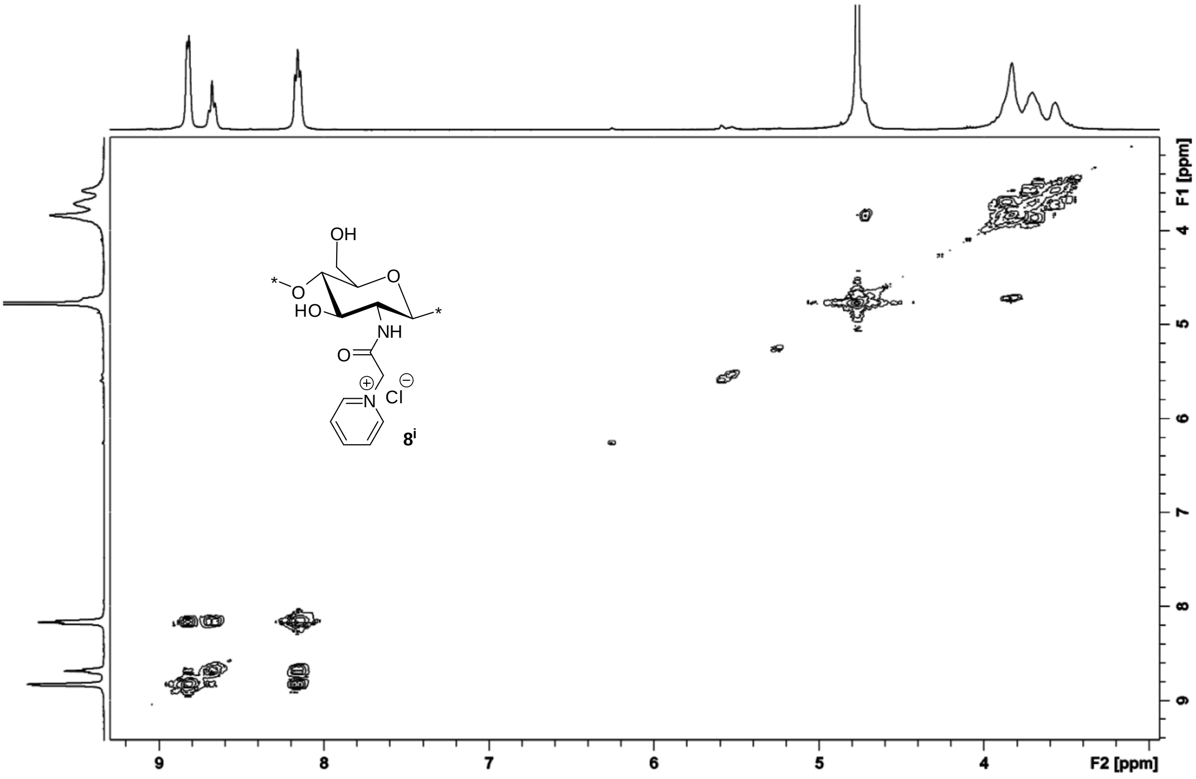

Figure S9. ^{1}H NMR (400 MHz, D_2O) spectrum of compound **6^{ii}** (TMA-CS^{ii}).

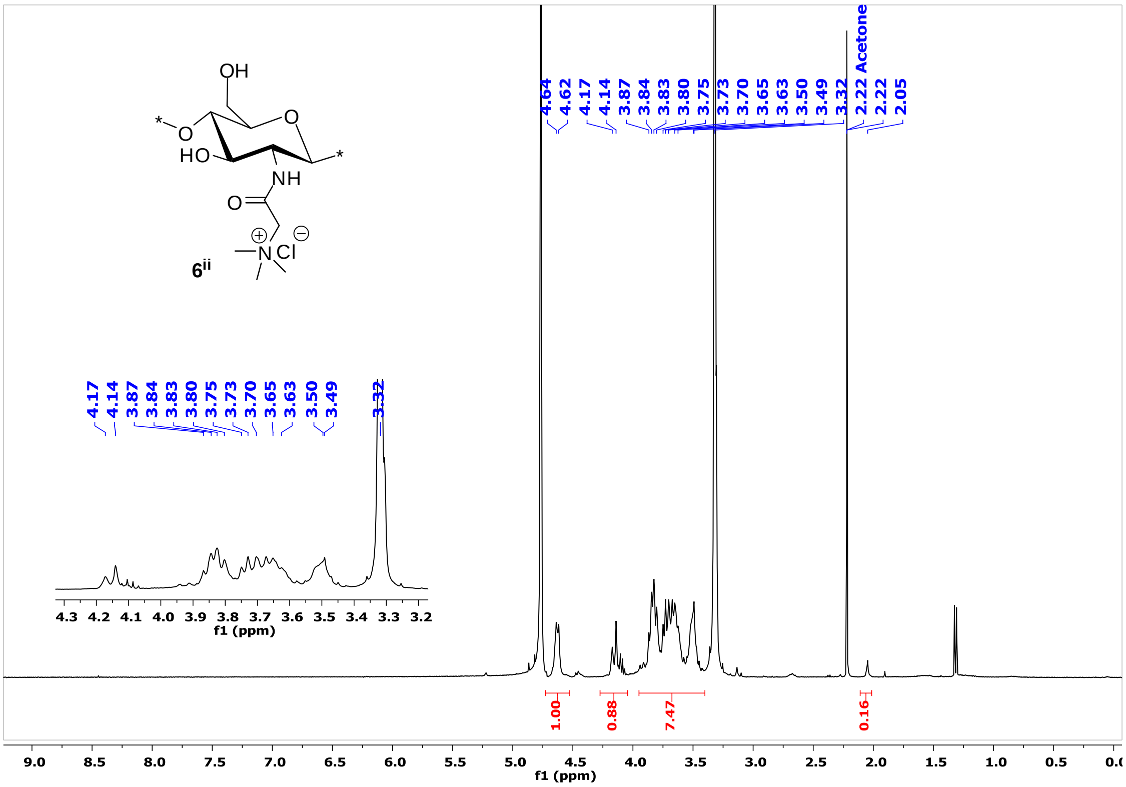

Figure S10. ^{1}H NMR (400 MHz, D_2O + 1 drop DCl) spectrum of compound **8^{ii}** (PyA-CS^{ii}).

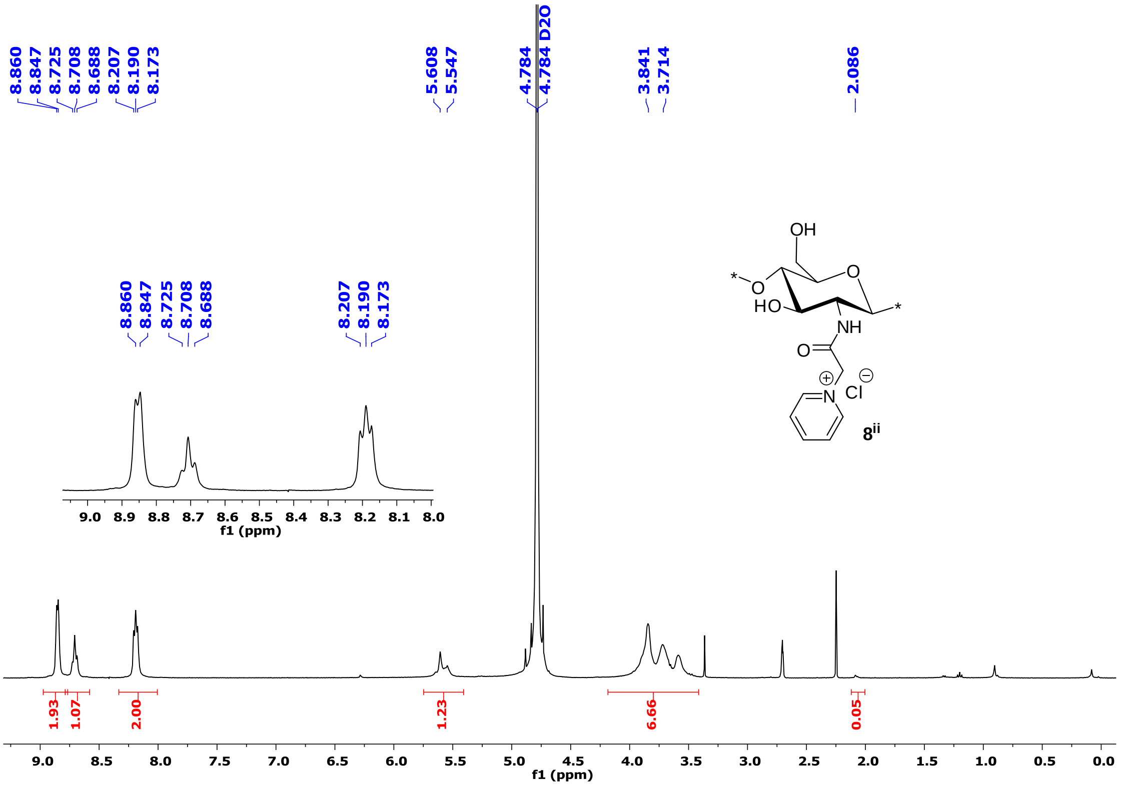

Figure S11. ^{1}H NMR (400 MHz, D_2O) spectrum of_compound **6^{iii}** (TMA-CS^{iii}).

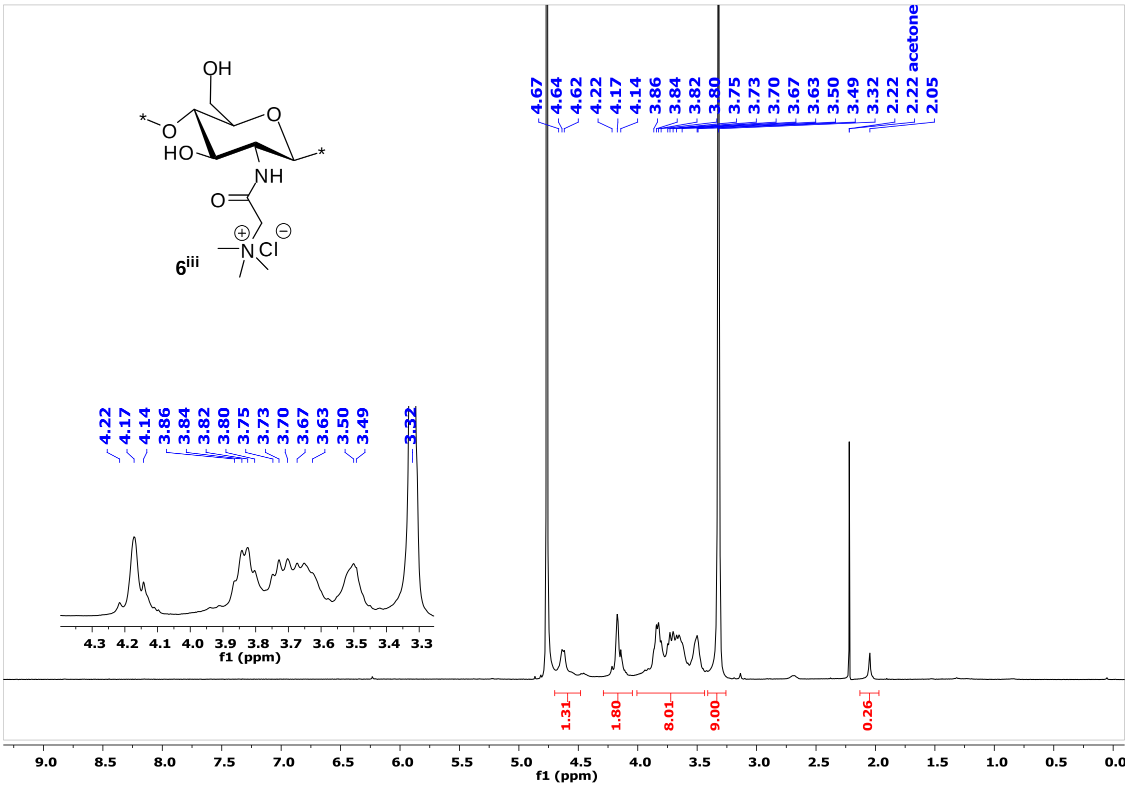

Figure S12. ^{1}H NMR (400 MHz, D_2O) spectrum of_compound **8^{iii}** (PyA-CS^{iii}).

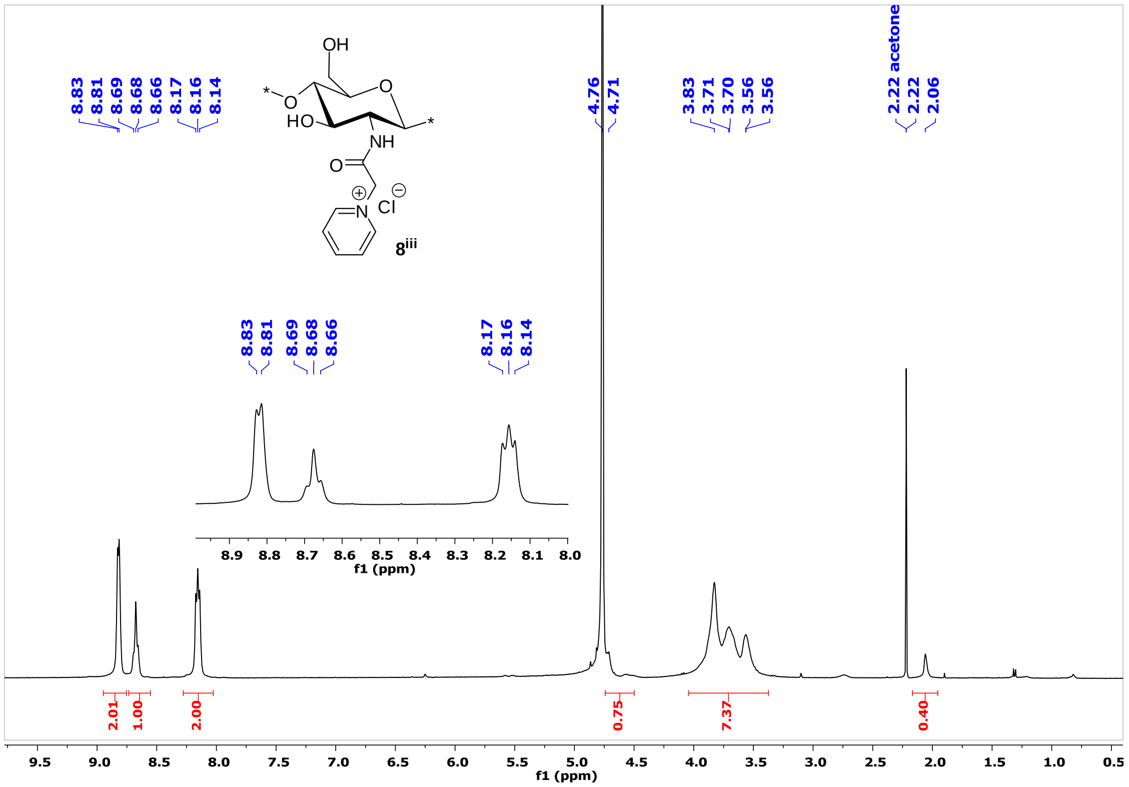

Figure S13. ^{1}H NMR (400 MHz, D_2O) spectrum of_compound **6**[iv] (TMA-CS[iv]).

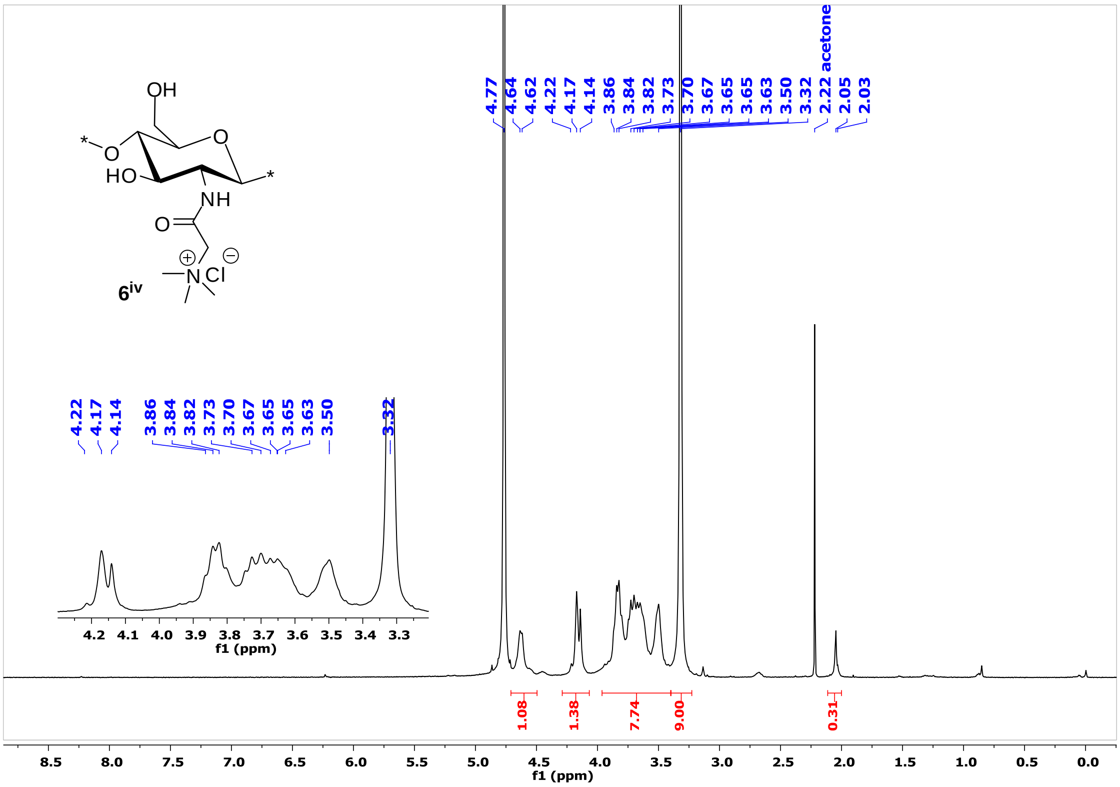

Figure S14. ^{1}H NMR (400 MHz, D_2O) spectrum of_compound **8**[iv] (PyA-CS[iv]).

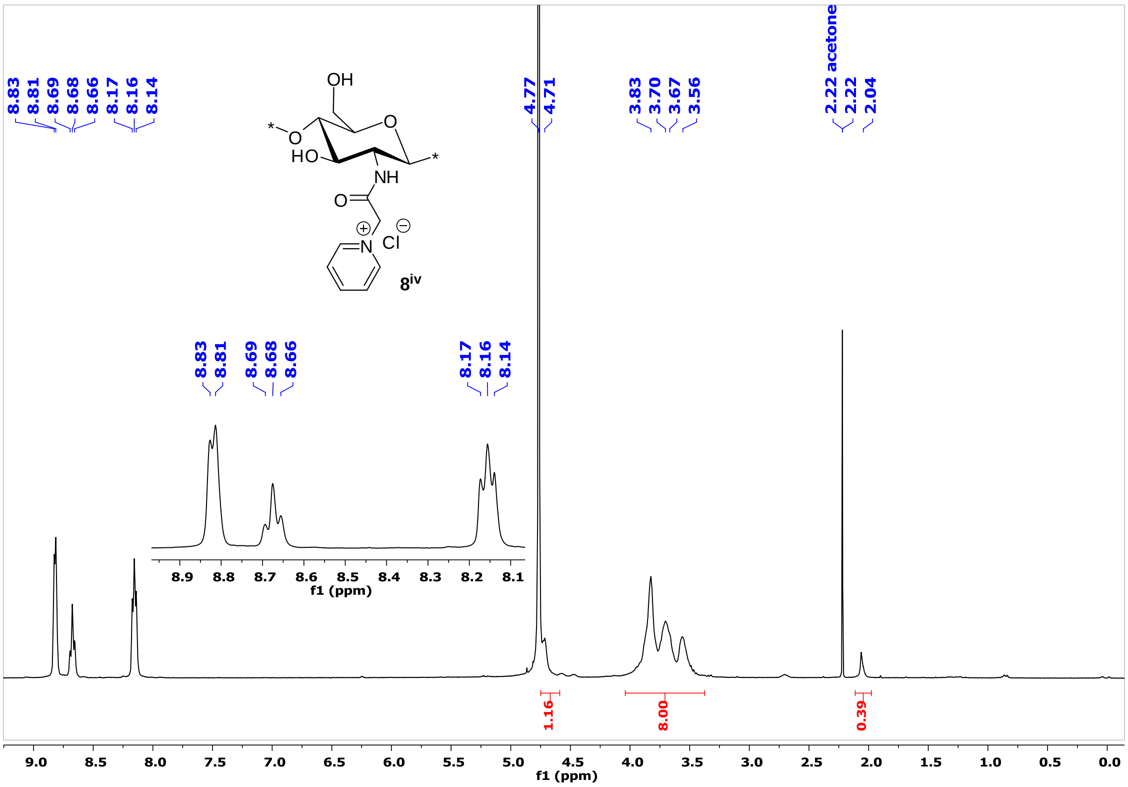

Figure S15. ^{1}H NMR (400 MHz, D_2O) spectrum of_compound **6^{v}** (TMA-CS^{v}).

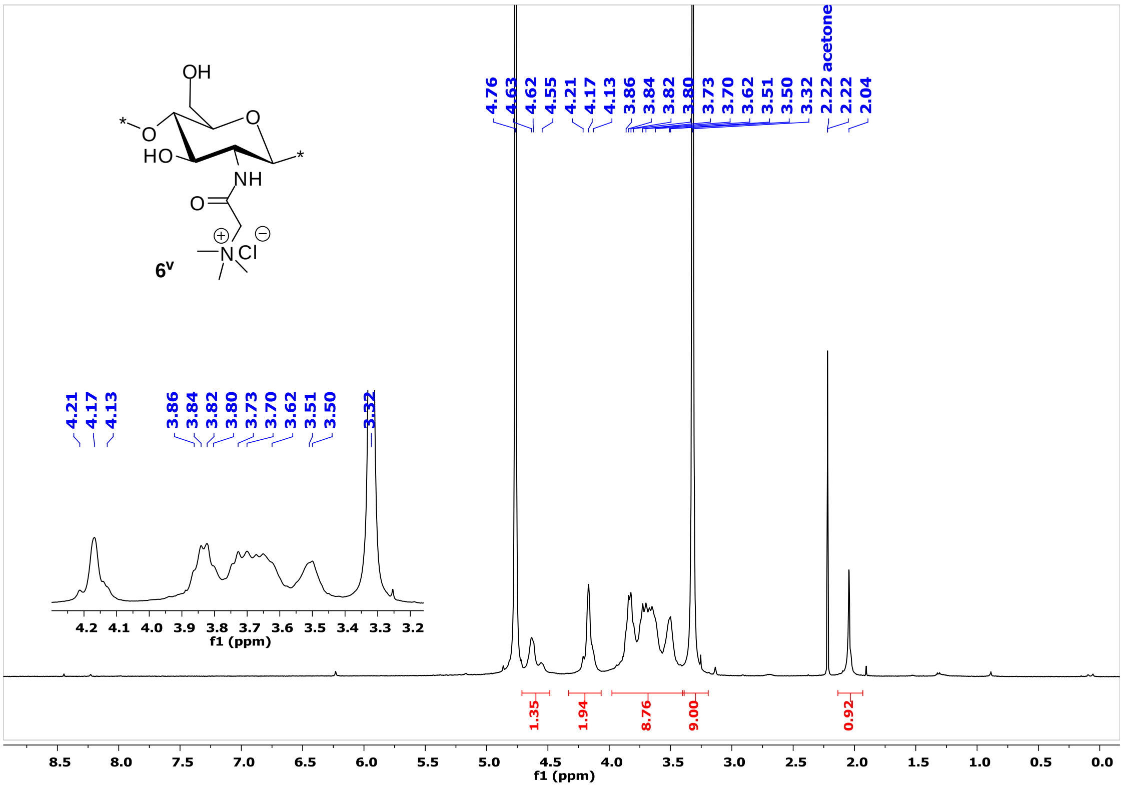

Figure S16. ^{1}H NMR (400 MHz, D_2O) spectrum of_compound **8^{v}** (PyA-CS^{v}).

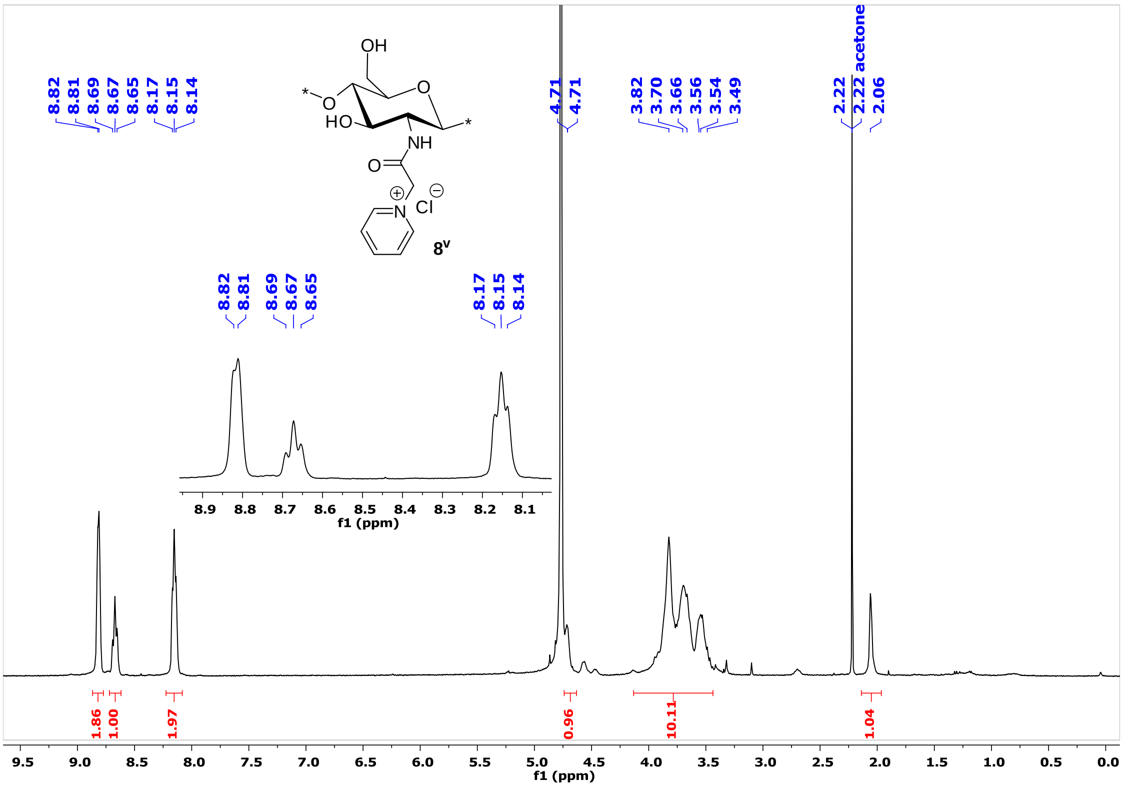

Figure S17. ^{1}H NMR (400 MHz, D_2O) spectrum of_compound **11**

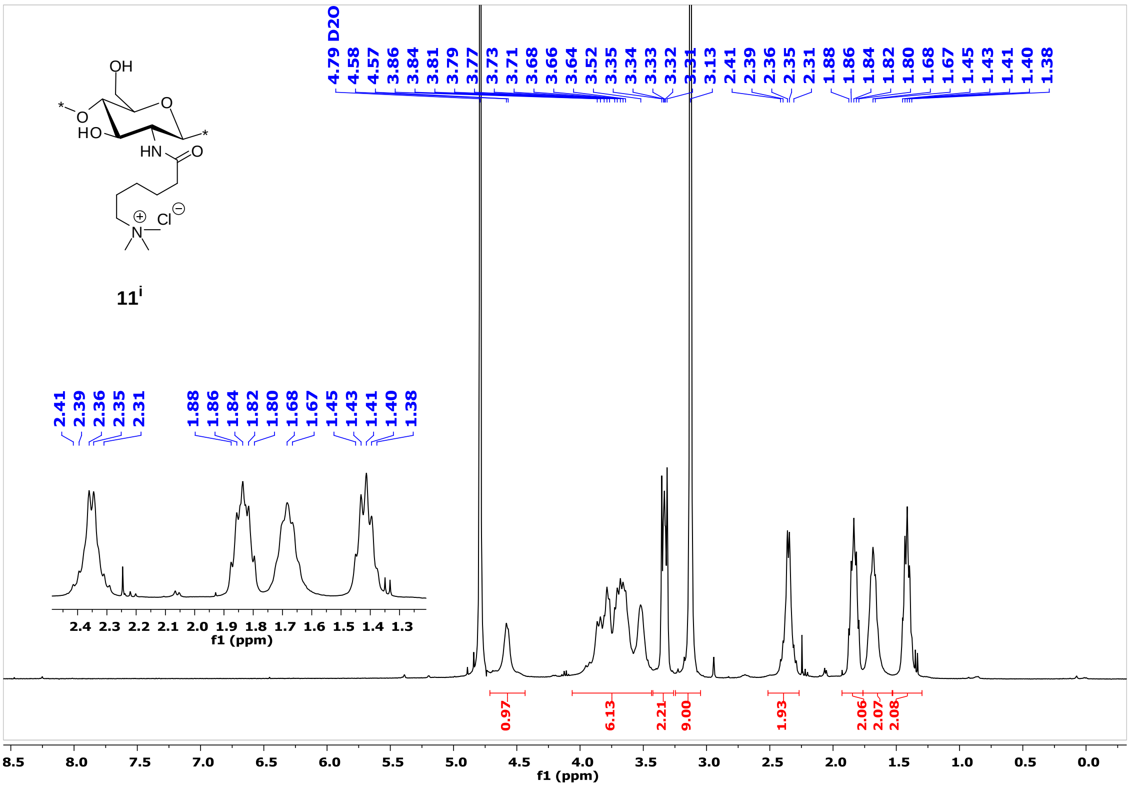

Figure S18. ^{1}H NMR (400 MHz, D_2O) spectrum of_compound **13^{i}** (PyHA-CS^{i}).

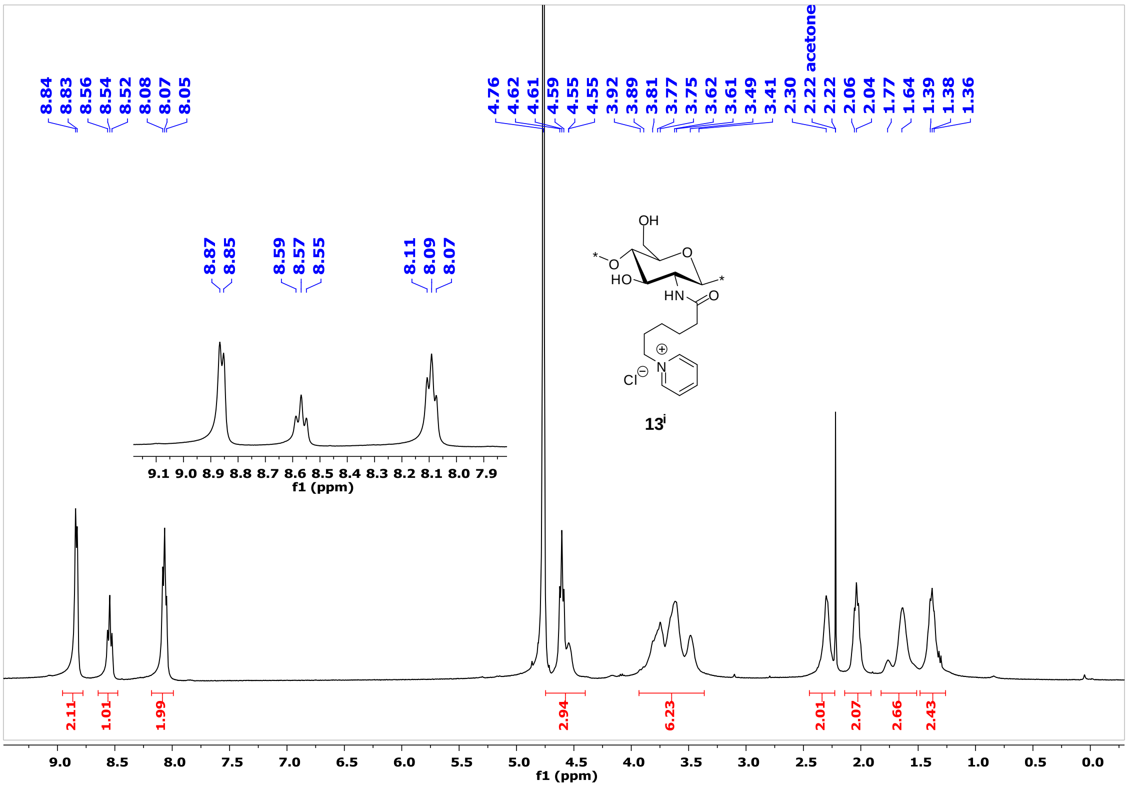

Figure S19. ^{1}H NMR (400 MHz, D_2O) spectrum of_compound **11^{ii}** (TMHA-CS^{ii}).

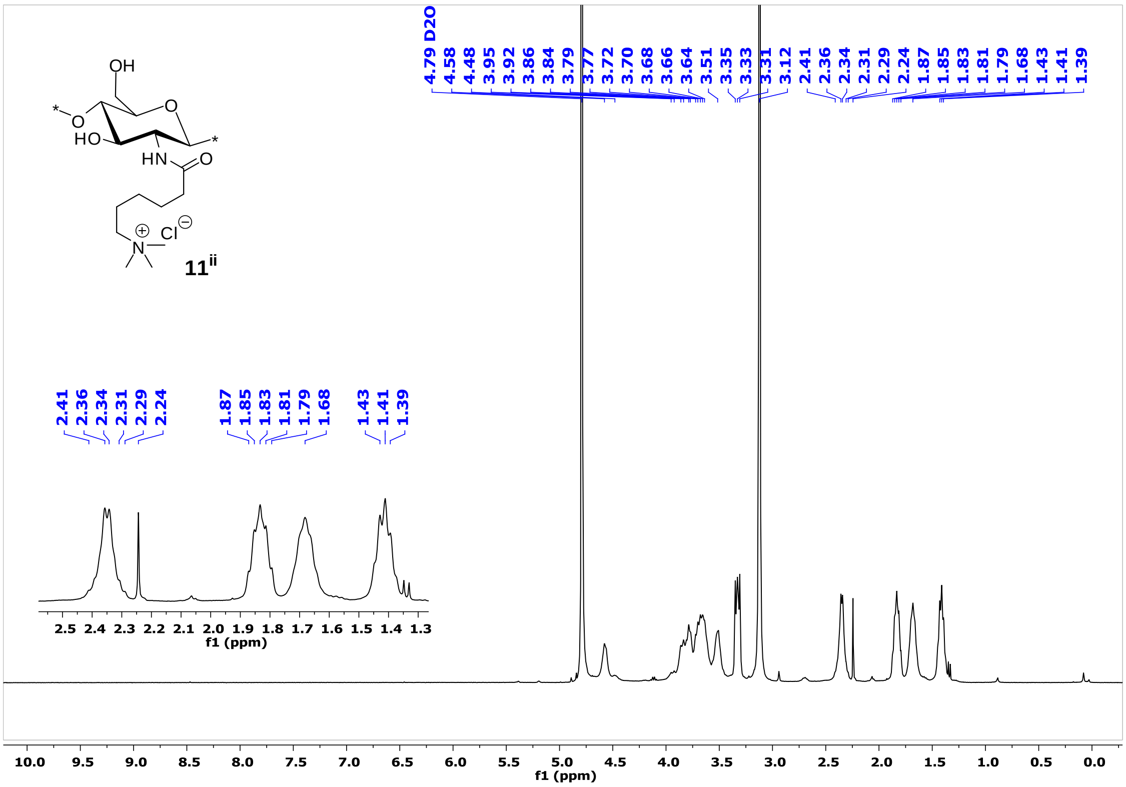

Figure S20.^{1}H NMR (400 MHz, D_2O) spectrum of compound **13^{ii}** (PyHA-CS^{ii}).

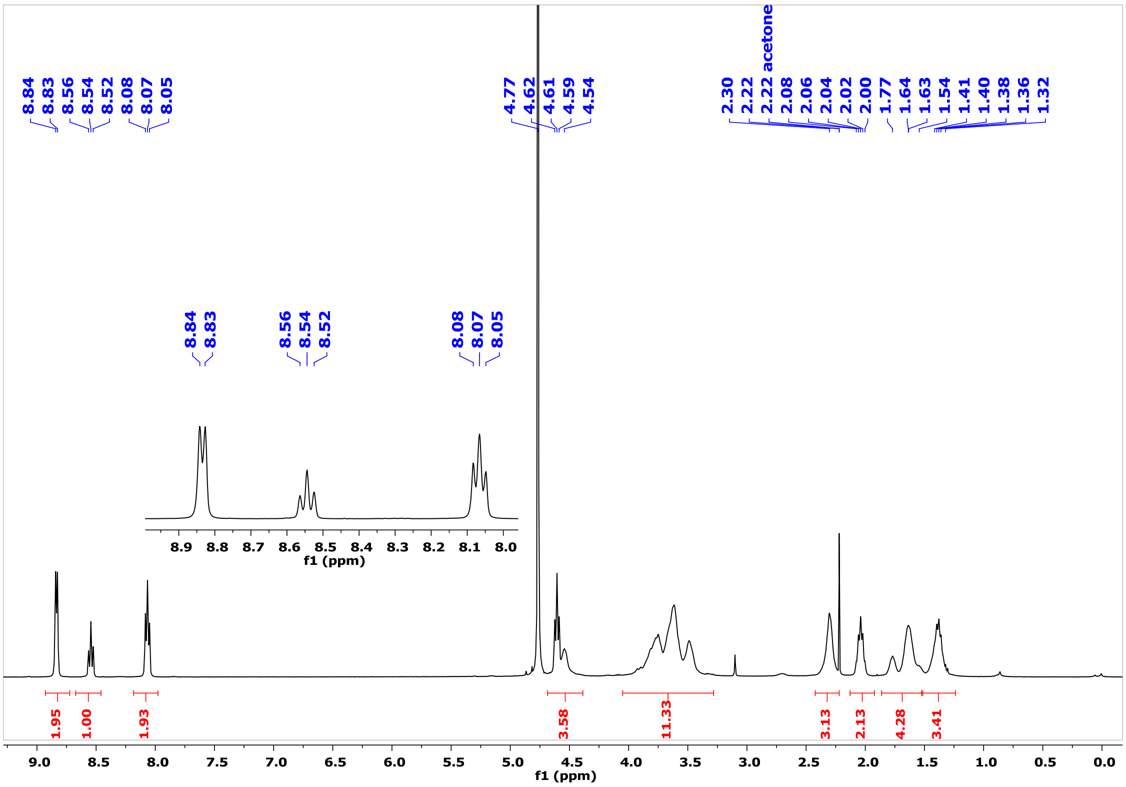

Figure S21. ^{1}H NMR (400 MHz, D_2O) spectrum of_compound **11**^{iii} (TMHA-CS^{iii}).

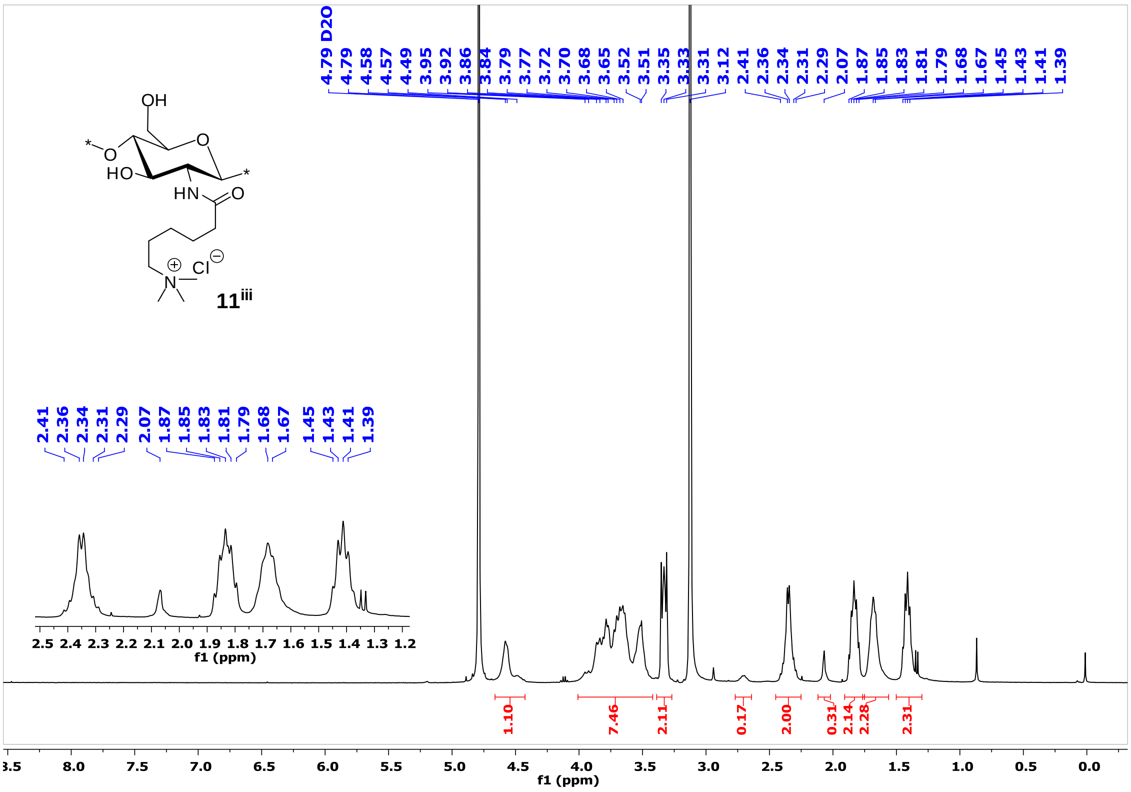

Figure S22. ^{1}H NMR (400 MHz, D_2O) spectrum of_compound **13**^{iii} (PyHA-CS^{iii}).

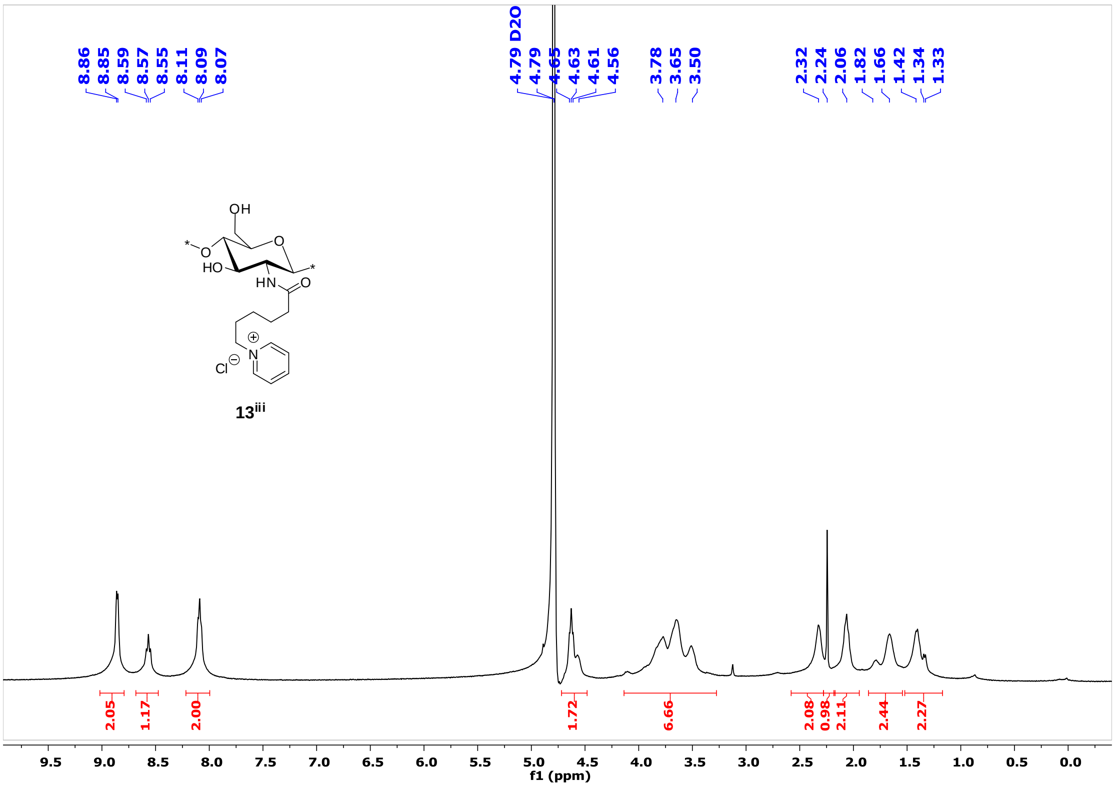

Figure S23. ^{1}H NMR (400 MHz, D_2O) spectrum of_compound **11**^{iv} (TMHA-CS^{iv}).

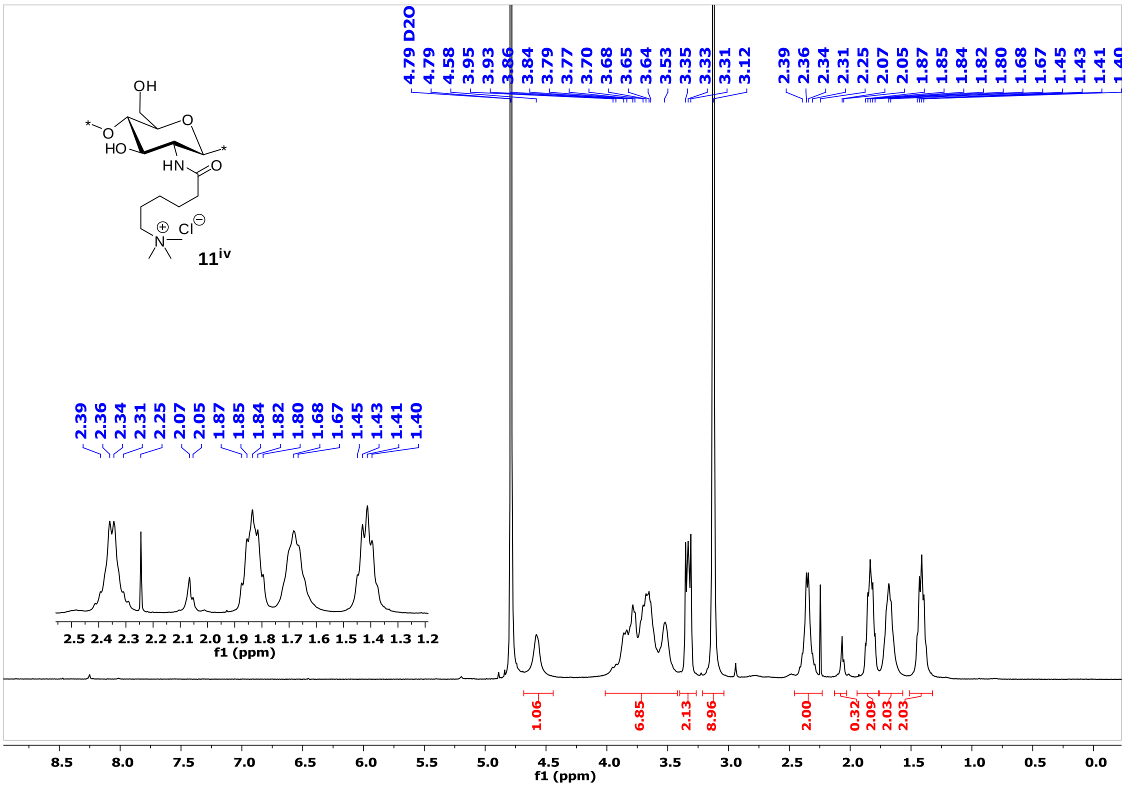

Figure S24. ^{1}H NMR (400 MHz, D_2O) spectrum of compound **13**^{iv} (PyHA-CS^{iv}).

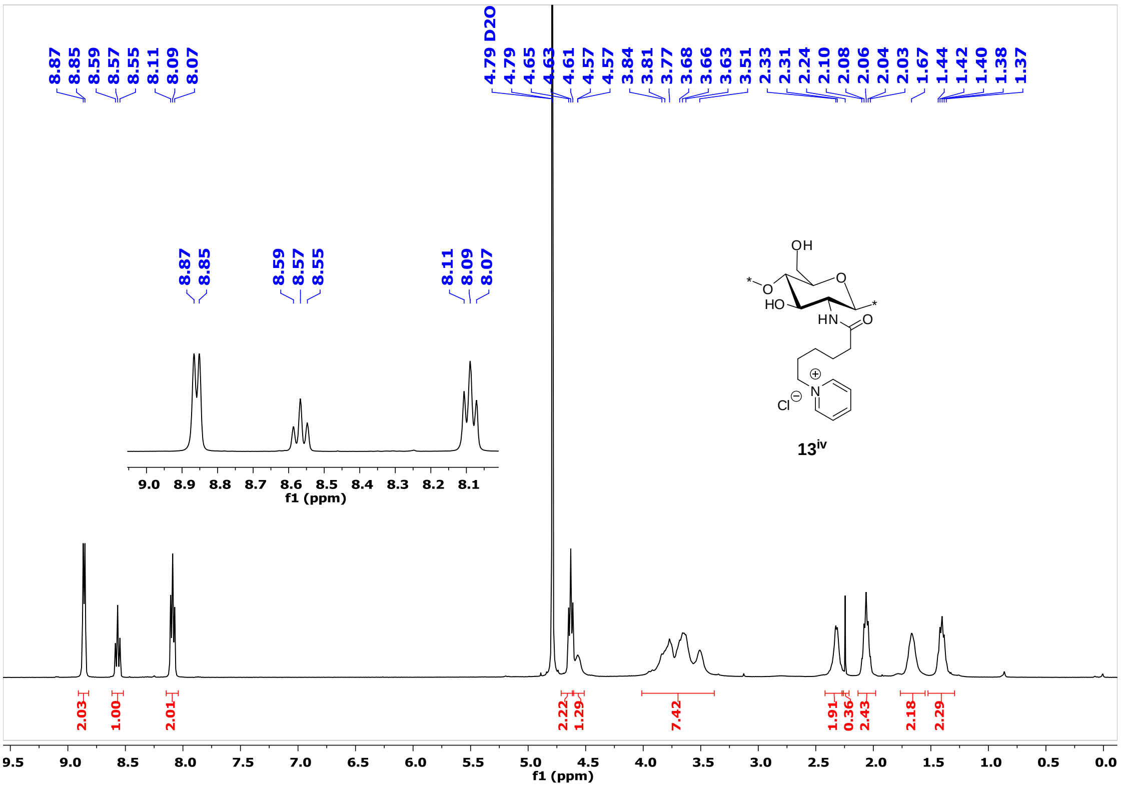

Figure S25. ^{1}H NMR (400 MHz, D_2O) spectrum of_compound **11**^{v} (TMHA-CS^v).

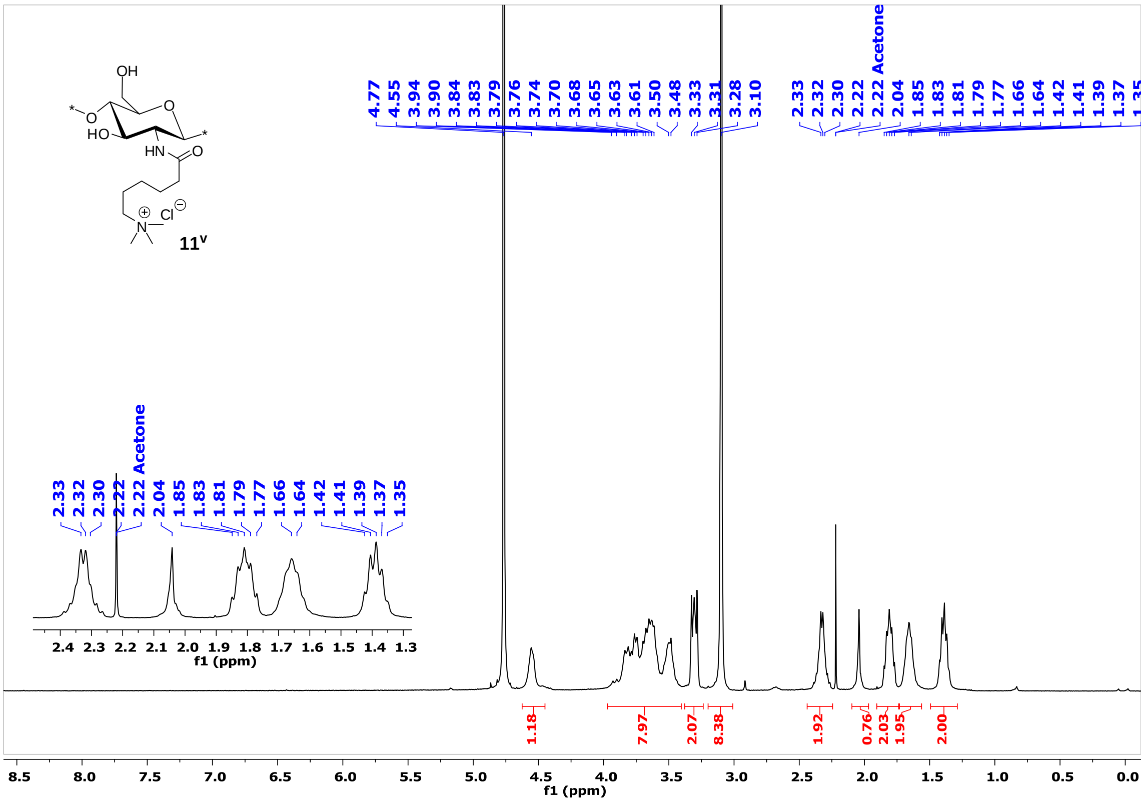

Figure S26. ^{1}H NMR (400 MHz, D_2O) spectrum of_compound **13**^{v} (PyHA-CS^v).

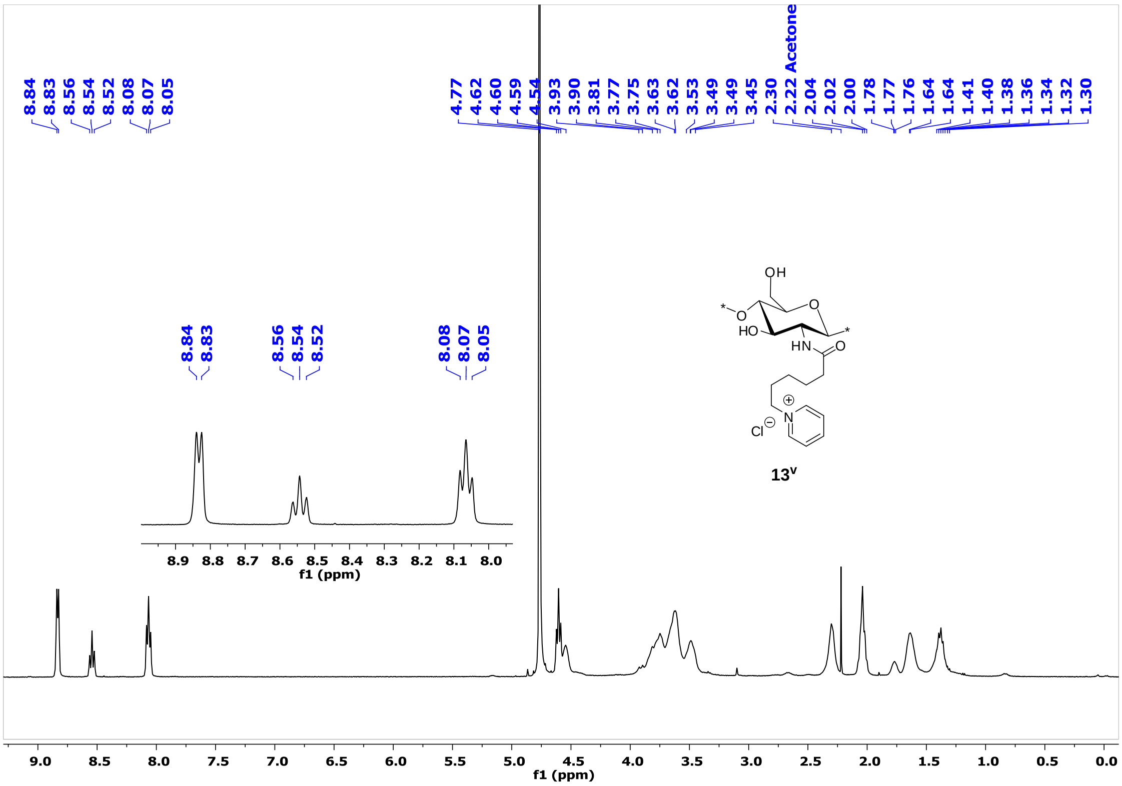

Figure S27. ^1H-^1H COSY (400 MHz, D_2O) spectrum of_compound **13^v** (PyHA-CS^v).

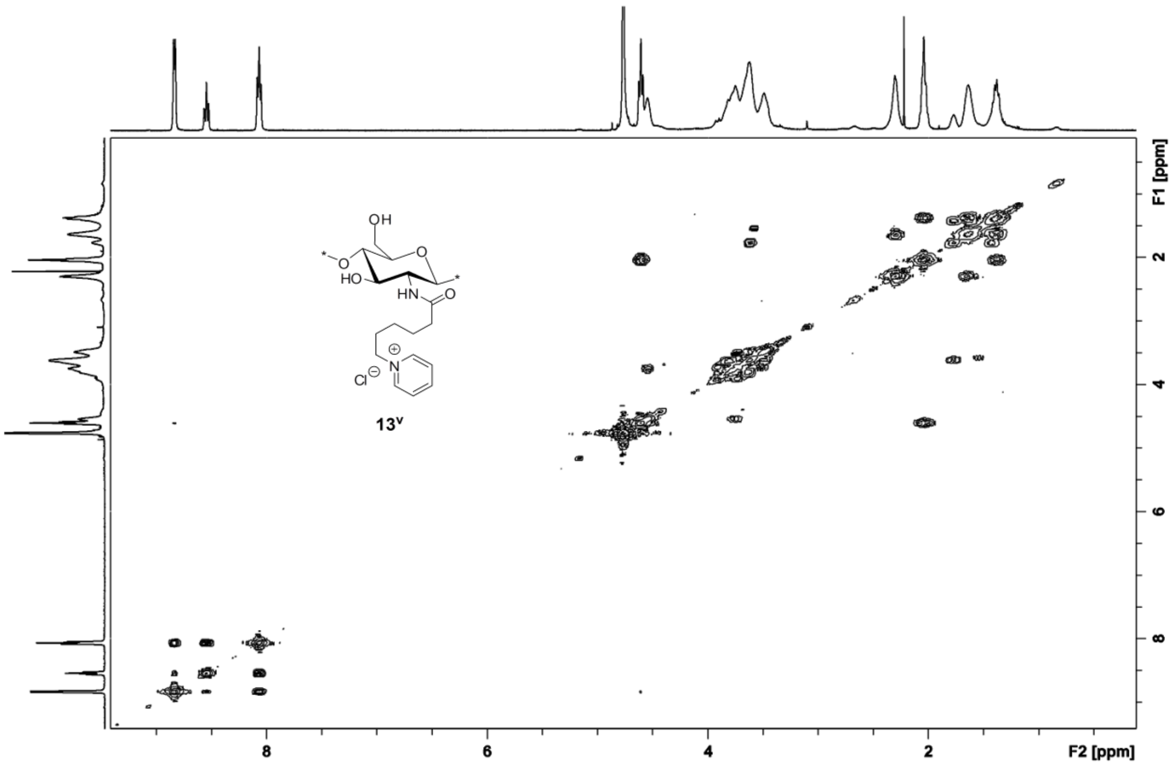

Figure S28. ^1H NMR (400 MHz, D_2O) spectrum of compound **15^i** (TMC^i).

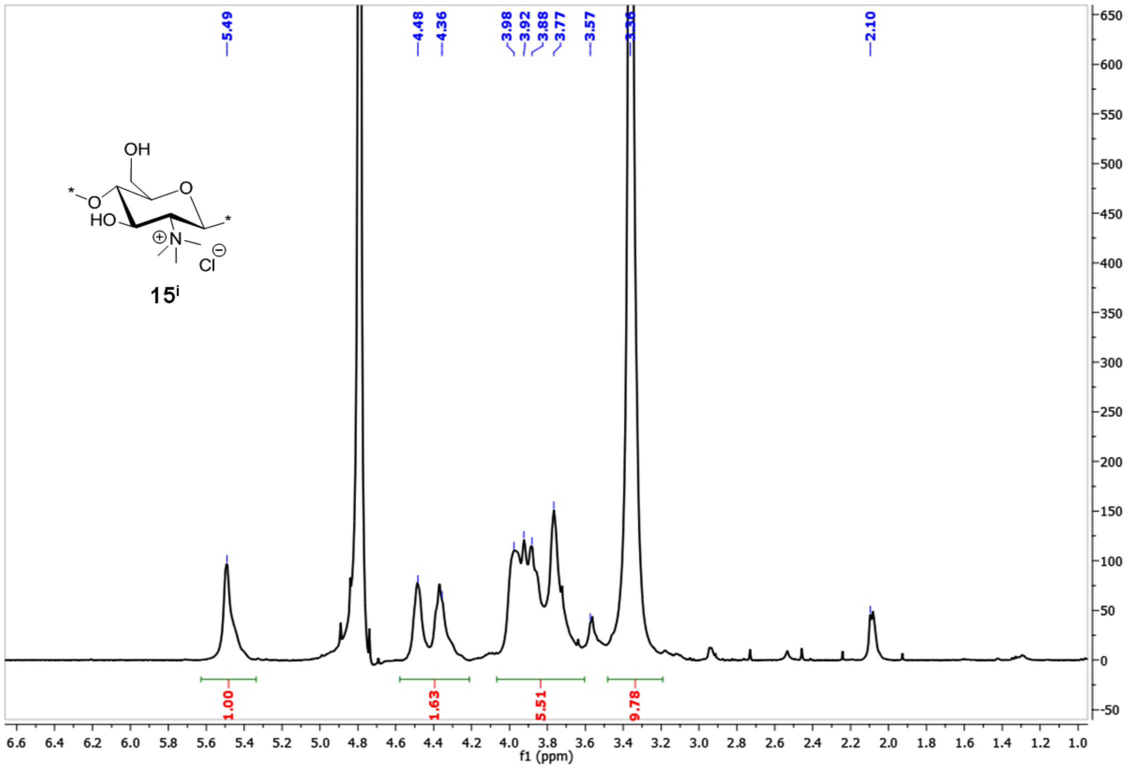

Figure S29. ^{1}H NMR (400 MHz, D_2O) spectrum of compound $\mathbf{15^{ii}}$ (TMC^{ii}).

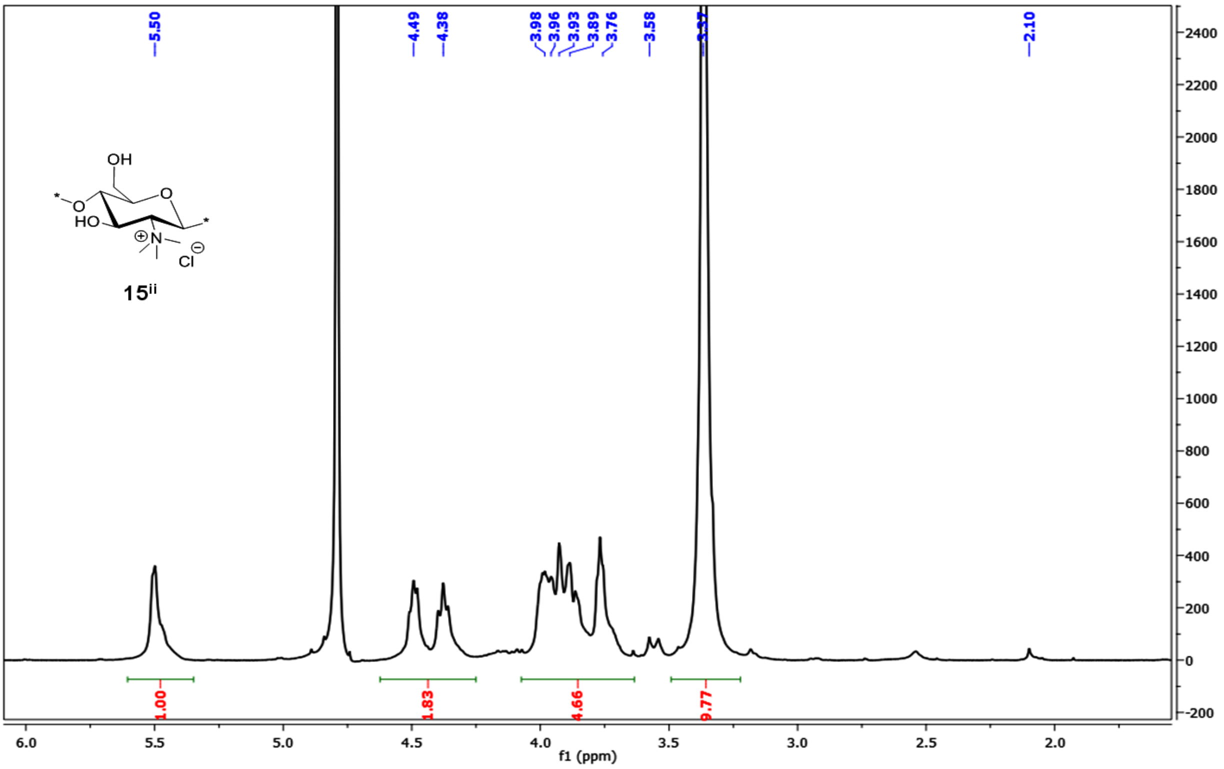

Figure S30. ^{1}H NMR (400 MHz, D_2O) spectrum of compound $\mathbf{15^{iii}}$ (TMC^{iii}).

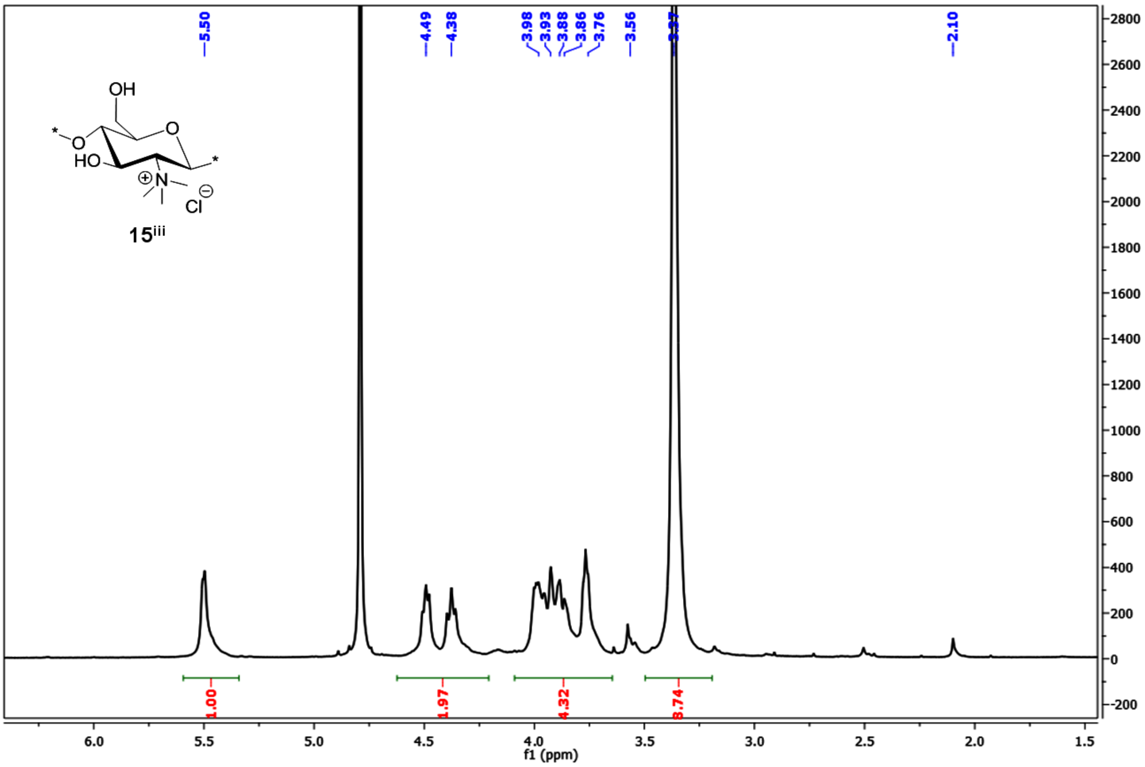

Figure S31. ^{1}H NMR (400 MHz, D_2O) spectrum of compound **15**

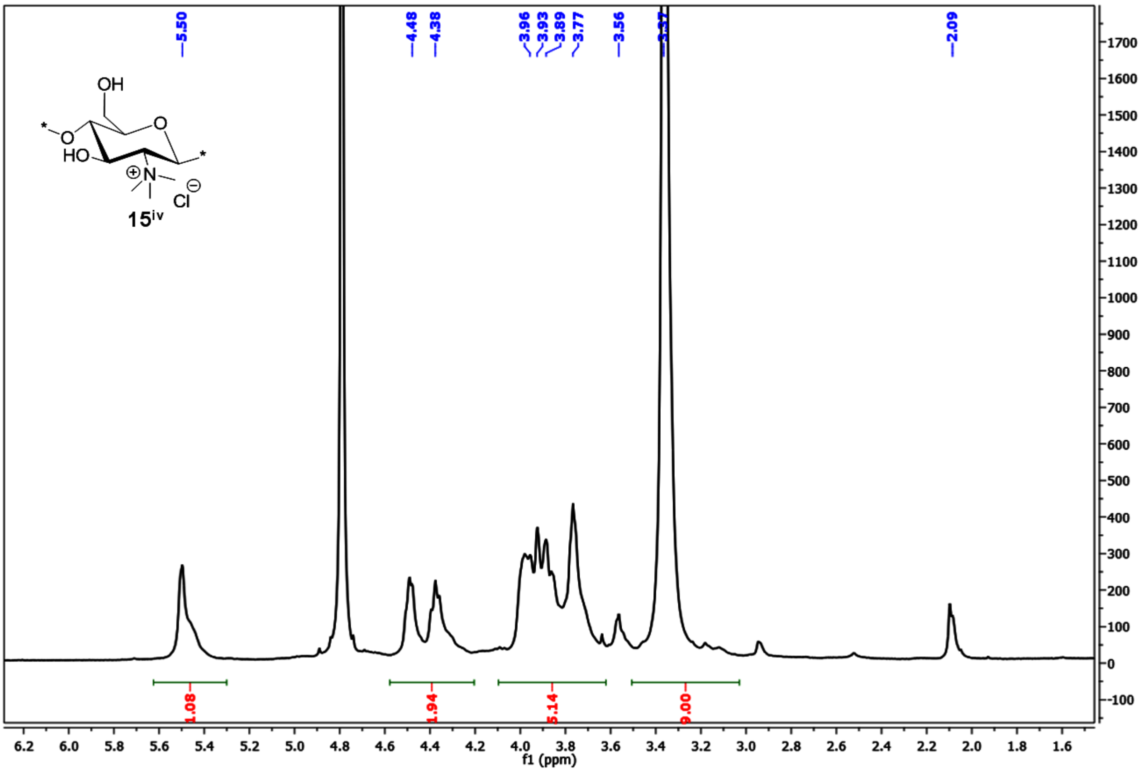

Figure S32. ^{1}H NMR (400 MHz, D_2O) spectrum of compound $\mathbf{15^{v}}$ (TMC^{v}).

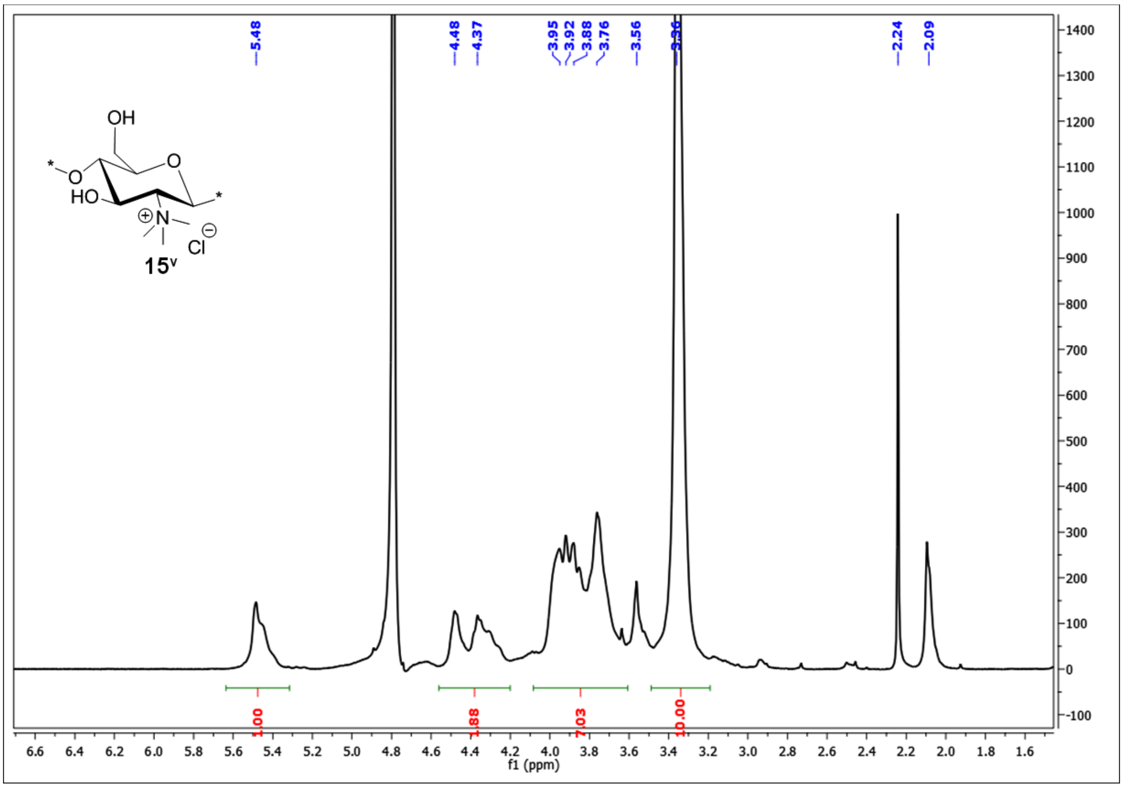

6. Graphs for Hemolysis Rate

Figure S33. Hemolysis rate (%) for Series $\mathbf{6}^{i\text{–}v}$ (TMA-chitosan$^{i\text{–}v}$).

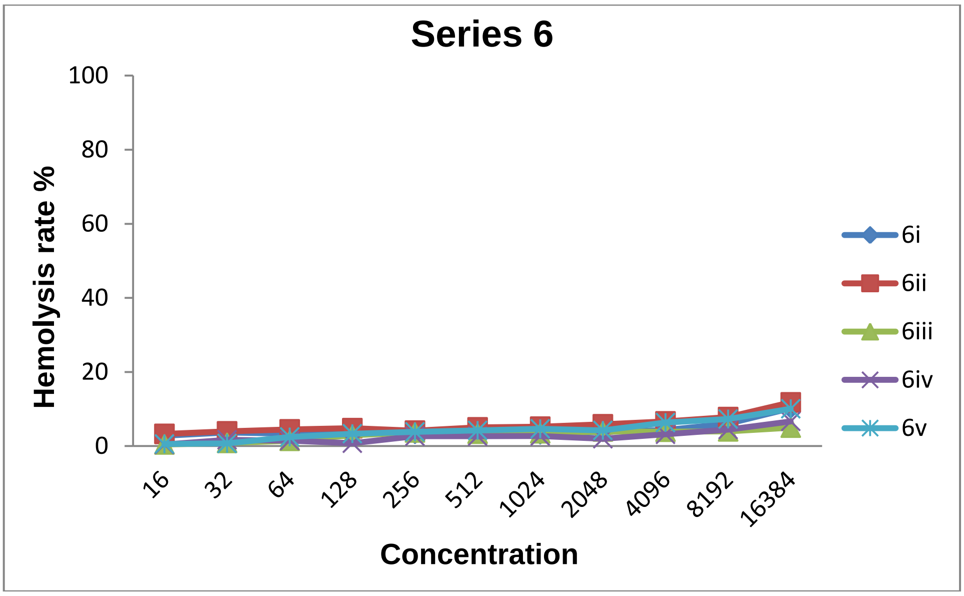

Figure S34. Hemolysis rate (%) for Series $\mathbf{8}^{i\text{–}v}$ (PyA-chitosan$^{i\text{–}v}$).

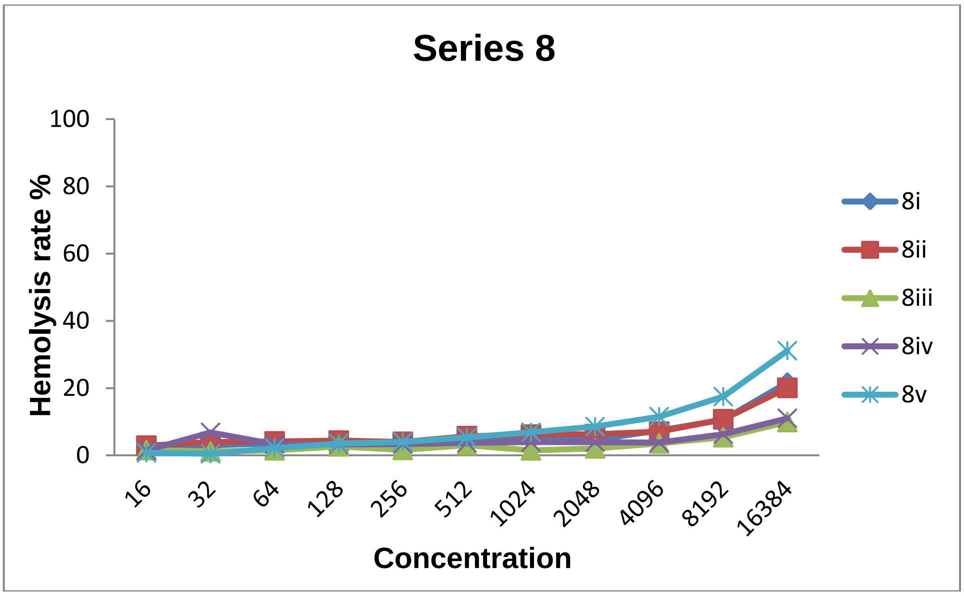

Figure S35. Hemolysis rate (%) for Series **11**$^{i–v}$ (TMHA-chitosan$^{i–v}$).

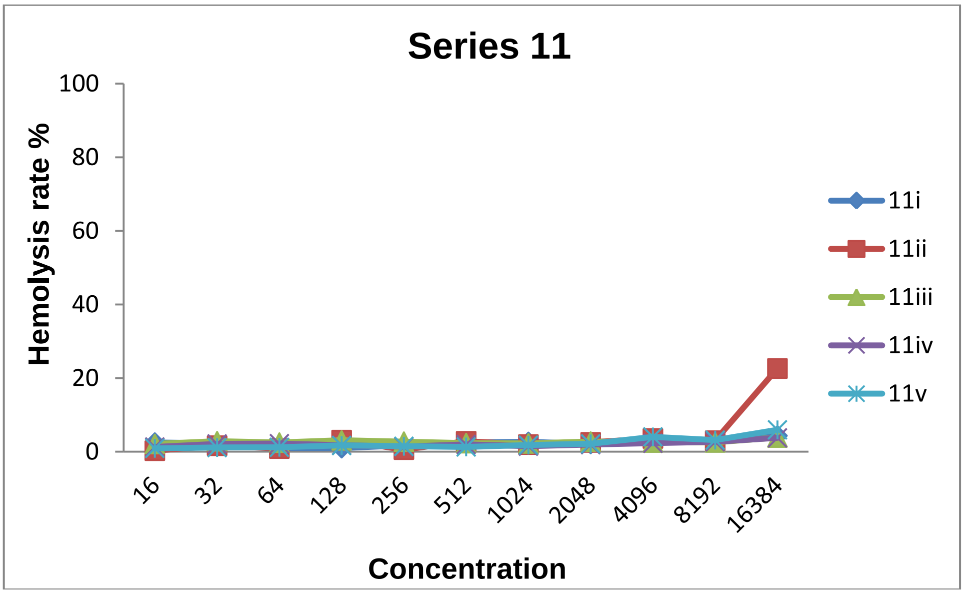

Figure S36. Hemolysis rate (%) for Series **13**$^{i–v}$ (PyHA-chitosan$^{i–v}$).

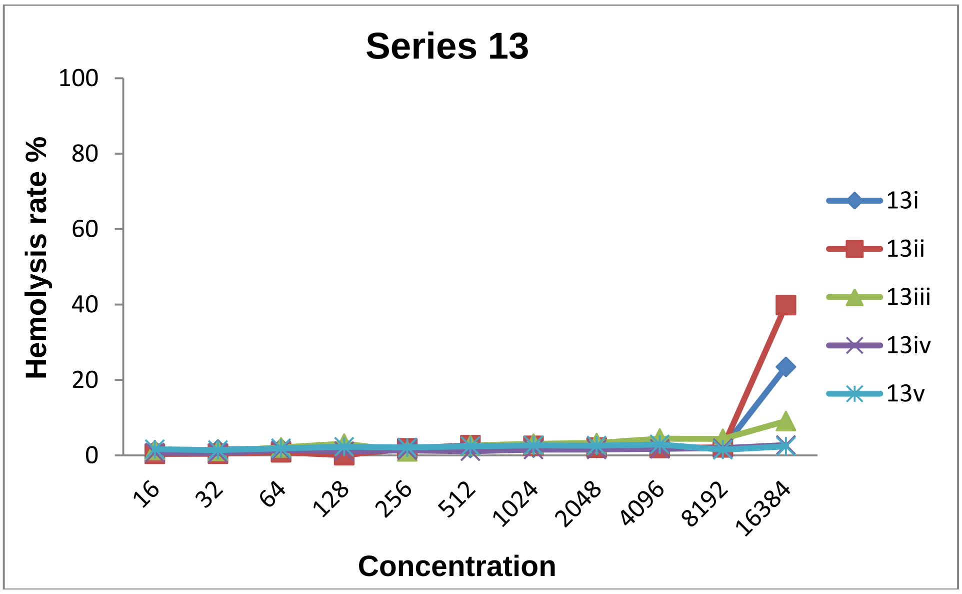

Figure S37. Hemolysis rate (%) for Series **15**$^{i–v}$ (TMC$^{i–v}$).

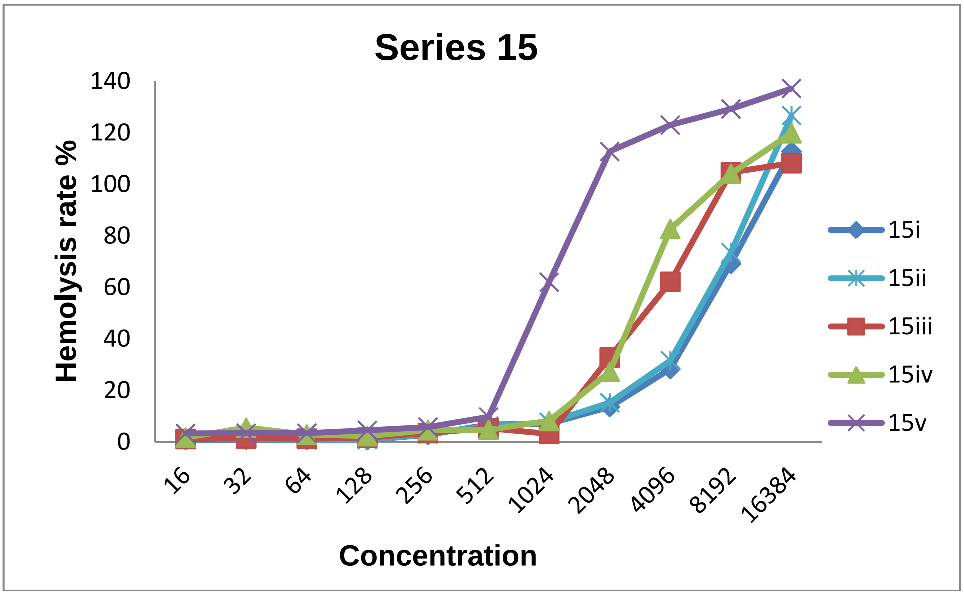

© 2014 by the authors; licensee MDPI, Basel, Switzerland. This article is an open access article distributed under the terms and conditions of the Creative Commons Attribution license (http://creativecommons.org/licenses/by/3.0/).
